# Photothermally Responsive Hydrogel Releases Basic Fibroblast Growth Factor to Promote the Healing of Infected Wounds

**DOI:** 10.34133/bmr.0156

**Published:** 2025-03-04

**Authors:** Shengnan Ma, Chengzhi Zhang, Xiaofeng Ren, Lei Song, Jiheng Shan, Yiming Liu, Siyuan Weng, Yang Wang, Dechao Jiao, Kewei Ren, Zhen Li, Xinwei Han, Yanan Zhao

**Affiliations:** ^1^ Department of Interventional Radiology, Key Laboratory of Interventional Radiology of Henan Province, The First Affiliated Hospital of Zhengzhou University, Zhengzhou 450052, China.; ^2^ Department of Endocrinology and Metabolism, The First Affiliated Hospital of Zhengzhou University, Zhengzhou University, Zhengzhou 450052, Henan, China.; ^3^ Henan Key Laboratory of Chronic Disease Prevention and Therapy & Intelligent Health Management, Zhengzhou 450052, Henan, China.; ^4^ Interventional Institute of Zhengzhou University, Zhengzhou 450052, China.; ^5^ Department of Information, The First Affiliated Hospital of Zhengzhou University, Zhengzhou, Henan 450052, China.

## Abstract

The treatment of infected wounds is often complicated by bacterial infection and impaired scar healing. Antibiotics and growth factors are typically utilized to address these clinical challenges and expedite wound healing. However, the use of hydrogels containing these therapeutic agents is often restricted to complex cases and increases treatment costs considerably. In this study, we developed a quaternized-chitosan-based hybrid hydrogel dressing (SQFB) with intrinsic antibacterial properties to address these limitations. The hybrid hydrogel contained interpenetrating polymer networks of basic fibroblast growth factor and black phosphorus nanosheets, facilitating a photothermal response that triggers the release of basic fibroblast growth factor upon near-infrared irradiation. In vitro experiments demonstrated that SQFB exhibits superior antibacterial, hemostatic, enhanced cell proliferation, and angiogenesis functions. Importantly, the results showed that SQFB can promote the healing of infected wounds by accelerating all 4 stages of wound repair while preventing scarring formation. RNA sequencing analysis revealed that combined treatment with SQFB and near-infrared irradiation can effectively modulate genes primarily associated with epithelial regeneration pathways and metabolic processes. Collectively, our findings suggest that this hybrid hydrogel holds great promise for the effective management of infected wounds.

## Introduction

The skin, as the largest and outermost organ of the human body, serves as the primary barrier against external environmental damage [1]. Skin lesions resulting from injuries, burns, surgeries, and other factors are commonly encountered in clinical practice. Wound healing is a complex, multiphase process that consists of 4 distinct stages: hemostasis, inflammation, proliferation, and remodeling [2]. When the integrity of the skin is compromised, it becomes susceptible to pathogen invasion. The release of toxins and subsequent inflammation can lead to wound infections, which markedly delay the healing process. Severe wound infections may result in invasive complications that pose a serious threat to health and well-being. Therefore, medical dressings are essential for wound treatment. However, traditional options such as gauze and cotton pads merely provide protective coverage, with no additional functional benefits. Hydrogels possess several advantages, including excellent biocompatibility that facilitates cell growth [3] and prevents the rapid degradation of encapsulated drugs [4], among others. Consequently, multifunctional antibacterial hydrogel wound dressings represent a substantial research trend.

In recent years, natural polysaccharides such as chitosan, cellulose, and alginate [5] have been utilized extensively in wound dressing applications. Notably, sodium alginate (SA) is an anionic polysaccharide that is rich in carboxyl (–COO) groups. Under mild conditions, the carboxyl group on the G-unit of SA can engage in electrostatic interactions with divalent cations (e.g., Ca^2+^, Cu^2+^, Mg^2+^, Fe^2+^, and Sr^2+^), leading to the formation of an “eggshell” structure. This interaction facilitates the stacking of G-units into a cross-linked network structure, promoting rapid hydrogel formation [6]. SA is inexpensive, easily sourced, and highly hydrophilic, and it exhibits low immunogenicity. Hence, it is suitable for various applications, including cell encapsulation and controlled drug release. Chitosan, the second most abundant polysaccharide in nature, can be modified using quaternary ammonium salts to produce quaternized chitosan (QCS). This modification not only enhances its solubility but also intensifies its positive charge, improving its bactericidal properties [7]. Studies conducted by Liang et al. [8] and Zhao et al. [9] demonstrated that QCS-based hydrogels possess both antibacterial and hemostatic effects, which make them valuable for wound healing.

Each stage of wound healing requires the involvement of various growth factors, such as epidermal growth factor, basic fibroblast growth factor (bFGF), and vascular endothelial growth factor [10]. bFGF is a single-chain polypeptide with multifaceted effects. This growth factor not only stimulates cell proliferation and angiogenesis in diverse cell types but also inhibits myofibroblast differentiation, thereby mitigating scar tissue formation [1,11]. Hence, commercially available local sprays containing bFGF are utilized in clinical therapy to accelerate wound healing [12]. However, the short half-life of bFGF and its susceptibility to degradation in vivo can lead to dilution and rapid metabolism upon direct local application. To overcome this limitation, Wang et al. [13] successfully integrated bFGF with copper-based metal–organic frameworks into a hydrogel material for wound repair. This innovative approach allowed for the sustained release of bFGF from the metal–organic frameworks into damaged skin areas under light stimulation, thereby enhancing the healing process.

Photothermal therapy involves the use of a photothermal agent that converts near-infrared (NIR) light energy into thermal energy. This noninvasive technique can provide broad-spectrum antibacterial activity and high selectivity [14]. Various photothermal agents such as metal nanostructure-based [15], carbon-based [16,17], phosphoro-based materials [18] are used to prepare hydrogels and applied to wound repair. Among them, black phosphorus nanosheets (BPNSs), which have a typical orthorhombic structure, serve as effective photothermal agents. Their ultrathin 2-dimensional honeycomb structure—combined with an exceptionally high specific surface area, high photothermal conversion efficiency, and superior biodegradability and biocompatibility—makes BPNSs suitable for a variety of biomedical applications, including photothermal therapy [19], photodynamic therapy [20], antibacterial therapy [21], and drug delivery [22]. In our previous study [23], BPNSs were utilized to fabricate nanofibers for the treatment of localized chronic wounds, and these nanofibers enabled the significant acceleration of wound healing. Furthermore, the combination of BPNSs with NIR irradiation resulted in the sustained and effective release of drugs, such as bFGF, loaded on the surface of the nanofibers.

**Fig. 1.**
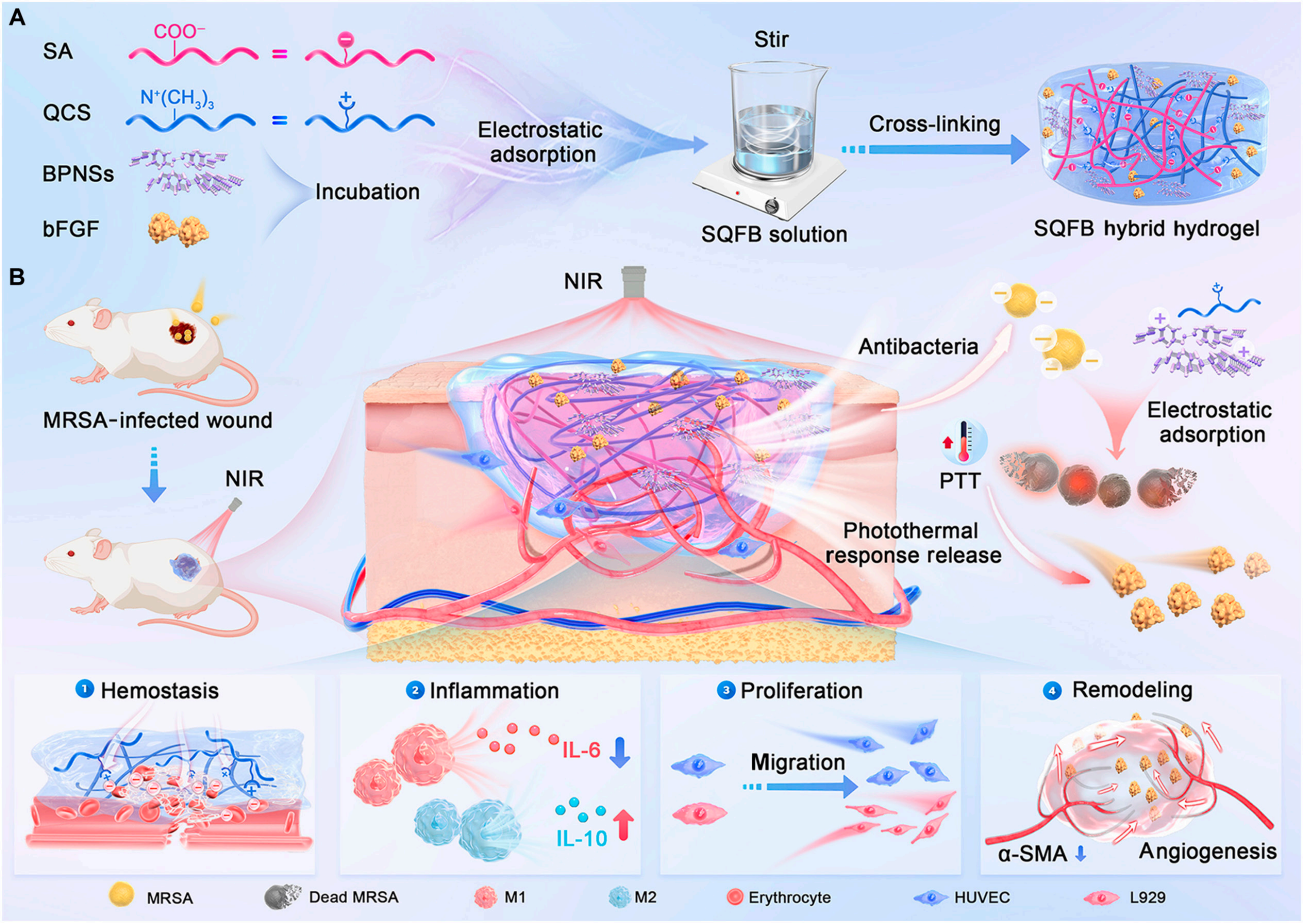
Schematic illustration of the preparation and mechanism of SQFB hydrogels. (A) Preparation of hybrid hydrogel containing basic fibroblast growth factor (bFGF) and black phosphorus nanosheets (BPNSs). (B) Mechanism of hybrid hydrogel at the wound site under near-infrared (NIR) response. SA, sodium alginate; QCS, quaternized chitosan; MRSA, methicillin-resistant *Staphylococcus aureus*; PTT, photothermal therapy; IL-6, interleukin6; IL-10, interleukin10; α-SMA, α-smooth muscle actin; HUVEC, human umbilical vein endothelial cell.

To address the challenges of treating infected wounds, in this study, we developed a photoresponsive hybrid hydrogel composed of SA, QCS, bFGF, and BPNSs. As illustrated in Fig. 1, the SQFB hybrid hydrogel was formed through electrostatic adsorption between SA and QCS, enabling the temperature-responsive release of bFGF in conjunction with QCS-mediated antibacterial action upon the NIR-irradiation-induced conversion of light energy into heat energy by BPNSs. This multifunctional hydrogel offered remarkable therapeutic effects, including accelerated wound hemostasis, reduced inflammation, enhanced cell proliferation and migration, and expedited vascular regeneration throughout all stages of the wound repair process while also preventing scar tissue formation. Owing to these exceptional characteristics, this hydrogel appears highly suitable for managing infected wounds and fulfills the requirements for advanced medical dressings.

## Materials and Methods

### Materials

SA and QCS were obtained from Aladdin Industrial Co. Ltd. (Shanghai, China), while BPNSs (0.8 mg/ml in ethanol solution) were sourced from XFNANO Materials Co. Ltd. (Nanjing, China). Recombinant human fibroblast growth factor (bFGF) was procured from Nanhai Longtime Pharmaceutical Co., Ltd. (Guangdong, China). Mouse fibroblasts (L929 cells) and human umbilical vein endothelial cells (HUVECs) were acquired from the Shanghai Cell Center, Chinese Academy of Sciences. Dulbecco’s modified Eagle medium (11965) and fetal bovine serum (S9030) were purchased from Solarbio Science & Technology Co. Ltd. (Beijing, China). Eagle’s minimum essential medium (320-006-CL) was obtained from WISENT Co. Ltd. (Nanjing, China). Penicillin–streptomycin 31 (V900929) and trypsin–EDTA (59417 C) were supplied by Sigma-Aldrich Trading Co. Ltd. (Shanghai, China). Cell Counting Kit-8 (CCK-8, CK04) was purchased from Dojindo China Co. Ltd. (Shanghai, China). LIVE/DEAD Viability/Cytotoxicity Kit (L3224) was obtained from Thermo Fisher Scientific Co. Ltd. (Shanghai, China), and the Bacterial Viability/Virulence Test Kit (L6060S) was sourced from BioScience Co. Ltd. (Shanghai, China). All other chemical and biological reagents were of analytical grade and were used without further modification.

### Preparation of hydrogels

First, 2 g of SA was dissolved in deionized water, and the mixture was stirred thoroughly using a magnetic stirrer to prepare a 2 wt% SA solution. Subsequently, 1 g of QCS was dissolved in deionized water using a magnetic stirrer to obtain a 1 wt% QCS solution. Finally, the SA and QCS solutions were mixed with each other in equal proportions. Pure NaCl was added to the mixture at a 3 wt% ratio and dissolved, followed by CaCl_2_ (1 M). The SQ hydrogel (S: SA; Q: QCS) was obtained through static molding. Subsequently, bFGF (final concentration: 300 μg/ml) was added to the SA and QCS mixture to obtain the SQF hydrogel. Similarly, BPNSs (final concentration: 0.5 mg/ml) were added to prepare the SQB and SQFB hydrogels (F: bFGF; B: BPNSs). Finally, the samples were freeze-dried using a freeze dryer to obtain a series of freeze-dried gels for subsequent experiments.

### Characterization of physicochemical properties

The surface structure of the hydrogel samples was analyzed using scanning electron microscopy (JSM-7401 F, JEOL, Japan). ImageJ (National Institutes of Health, Bethesda, USA) was employed to measure the mean pore size in each hydrogel sample. The chemical structure of the hydrogels was assessed using Fourier transform infrared spectroscopy (FTIR; TNZ1-5700, Nicolet, USA) in the scanning range of 400 to 4,000 cm^−1^ to analyze functional group vibrations. Raman spectroscopy (RAMANforce, Nanophoton, Japan) was utilized to assess chemical bond vibrations in the range of 100 to 3,600 cm^−1^. Further, dry cylindrical samples of the hydrogels (5 mm × 5 mm, diameter × height) were immersed in water, and their water absorption rate was measured at fixed time intervals. The mechanical properties of the hydrogels were evaluated using a universal testing machine (CMT 6503, Shenzhen SANS Test Machine, China) through compression testing at room temperature (25 °C). Briefly, the hydrogels were fabricated into a cylindrical shape (10 mm × 8 mm, diameter × height) and compressed at a strain rate of 2 mm/min, with the maximum compressive strain set at 80%.

### Assessment of photothermal properties

To evaluate their photothermal properties, the hydrogel samples were subjected to 808-nm laser irradiation at power densities of 0, 0.5, and 0.8 W/cm^2^ for 5 min. The photothermal stability of SQFB was evaluated during 5 heating cycles under laser irradiation (808 nm, 0.8 W/cm^2^, 2 min), followed by free cooling cycles. Additionally, the photothermal properties were also evaluated using NIR laser irradiation in an in vivo mouse wound model. The wounds were covered with hydrogel samples, which were subsequently irradiated using 808-nm NIR lasers at varying power densities (0.5 and 0.8 W/cm^2^). The temperature changes during the experiment were monitored in real time using a Testo 865 thermal imaging camera (Testo Instruments International Trading Co., Ltd., Shanghai, China).

### In vitro drug release behavior

For the in vitro drug release assay, SQF and SQFB samples were immersed in 5 ml of phosphate buffer saline (PBS) and incubated at room temperature, with or without 808-nm NIR irradiation at power densities of 0.5 and 0.8 W/cm^2^. The NIR irradiation was applied for 10 min after 8-h intervals. All suspensions were maintained under continuous oscillation at 50 rpm. At predetermined time points, 1.0 ml of the release medium was collected for analysis, and an equal volume of fresh PBS was added to the extraction vials. Subsequently, bFGF release was analyzed using a bFGF-specific enzyme-linked immunosorbent assay (ELISA) kit (Fine Biotech Co., Ltd., Wuhan, China) at different time points (0 to 7 d) based on the absorbance at 450 nm.

The skin penetration of the drug was assessed based on the ion penetration method using a pigskin model. First, all pigskin samples were assessed for their integrity based on standard protocols. Skin samples (with an exposed surface area of 1.0 cm^2^) were affixed to a modified Franz cell and coated with the SQFB hydrogel (1 mm in thickness). Some of these samples were exposed to NIR irradiation at a power density of 0.8 W/cm^2^, while others were left untreated. The receiving cell was filled with 2.0 ml of PBS buffer (pH = 7.3 to 7.4). Subsequently, electrodes were connected to both the skin samples and receiver cells, and a current of 0.42 mA was applied across the electrodes for 3 h. The buffer in the receiver cells was replaced periodically, and the drug penetration in the skin was quantified by measuring the bFGF release from the samples using ELISA kits [24].

### Assessment of biocompatibility

#### Hemolysis rate

Whole blood was collected from rabbit hearts and diluted with normal saline at a ratio of 1:1.25. The hydrogels were incubated with the diluted blood samples for 1 h at 37 °C, and the blood samples were then centrifuged at 3,000 rpm for 10 min. The optical density (OD) of the supernatant was measured at 545 nm using a microplate reader (BioTek, USA). The hemolysis rate was calculated using the following formula: hemolysis rate (%) = (OD_s_ − OD_n_)/(OD_p_ − OD_n_) × 100, where OD_s_ represents the OD value of the sample, OD_p_ is the OD value of the positive control, and OD_n_ is the OD value of the negative control.

#### Live/dead cell staining

Cell viability was assessed using a live/dead cell double-staining kit. HUVECs and L929 cells (5 × 10^4^ cells/ml) were seeded in separate 24-well plates and incubated overnight. The cells were divided into the blank control (B.C.), SQ, SQB + NIR, SQF, SQFB, and SQFB + NIR groups. An equal volume of PBS or hydrogel extract was added to the wells corresponding to each experimental group. The samples in the NIR groups were exposed to 808-nm NIR irradiation (0.8 W/cm^2^) at 8-h intervals. After 24 h of incubation, the cells were washed with PBS. The cells were then stained with calcein-AM and propidium iodide for 20 min. This was followed by imaging using a fluorescence microscope (Carl Zeiss, Germany). The ImageJ software was employed to analyze the number of live and dead HUVECs and L929 cells.

#### CCK-8 assay

Cell proliferation assays were conducted using the CCK-8 kit. HUVECs and L929 cells (1 × 10^3^/ml) were seeded in separate 96-well plates and incubated overnight. An equal volume of PBS or hydrogel extract was added to the wells corresponding to each experimental group. Over a period of 1 to 3 d, the CCK-8 reagent was added to each well. The cells were incubated with this reagent at 37 °C under 5% CO_2_ for 3 h to facilitate color development. The OD was then measured at 450 nm using a microplate reader. The relative cell proliferation rate was calculated as follows: Relative cell viability (%) = [(OD_s_ − OD_b_)/(OD_c_ − OD_b_)] × 100. Here, OD_b_ represents the OD value of the blank control, OD_c_ denotes the OD value of the control group at a specific time point, and OD_s_ denotes the OD value of the sample group at a specific time point.

#### Scratch assay

HUVECs and L929 cells (1 × 10^5^/ml) were seeded into 6-well plates. Once a confluent cell monolayer was established, a sterile P200 pipette tip was used to create a scratch in this monolayer. The wells were rinsed with PBS to remove any floating cells, and the cells were treated with the treatment factors corresponding to their respective experimental groups. The HUVECs and L929 cells were imaged under a light microscope at 0 and 24 h. The wound healing rate was calculated using the following formula: wound healing rate (%) = (*W*_0_ − *W*_24_)/*W*_0_ × 100, where *W*_0_ is the wound area at 0 h and *W*_24_ is the wound area at 24 h.

#### Transwell assay

HUVECs and L929 cells (5 × 10^4^/ml) were seeded into the upper chamber of a Transwell system, and the appropriate treatment factors were added to the lower chamber. After 24 h, a cotton swab was used to gently remove any nonmigratory cells that remained on the upper surface of the filter membrane. The migratory cells on the lower surface of the filter membrane were subsequently fixed with 4% paraformaldehyde for 20 min. Following fixation, the cells were stained with 0.5% crystal violet and imaged under a light microscope. The ImageJ software was utilized for quantifying the number of migrating cells in each group.

#### Tube formation assay

First, 200 μl of matrix gel (BD Biosciences, Franklin Lakes, NJ, USA) was added to the lower chamber of a pre-cooled 24-well plate and allowed to polymerize at 37 °C for 30 min. Subsequently, a suspension of HUVECs (2 × 10^4^ cells/ml) was added onto the polymerized matrix gel. The cells were subsequently treated with the treatment factors corresponding to their respective experimental groups. After 6 h, the cells were stained with calcein-AM and imaged under a fluorescence microscope (Carl Zeiss, Germany). The branch points and lengths of the capillary-like structures were quantified using the ImageJ software.

### Evaluation of antibacterial performance

The antibacterial activity of the hydrogels against methicillin-resistant *Staphylococcus aureus* (MRSA) and *Escherichia coli* was evaluated. The bacterial samples were divided into 6 groups and treated with the corresponding treatment agents: B.C., P.C., SQ, SQF, SQFB, and SQFB + NIR. The hydrogels were exposed to ultraviolet light for 30 min to ensure complete sterilization prior to subsequent application. Bacterial colony formation assays, bacterial proliferation assays, and live/dead bacterial staining assays were performed to evaluate the survival and proliferation capabilities of the treated bacteria.

#### Bacterial colony formation assay

Sterile hydrogels were placed in each well of a 6-well plate, with PBS added to the B.C. group and targeted antibiotics added to the P.C. group. First, the *E. coli* and MRSA suspensions (1 × 10^8^ colony-forming units [CFU]/ml) were treated with SQ, SQF, SQFB, and SQFB + NIR for 5 h in a relatively humid environment. The SQFB + NIR group was exposed to 808-nm NIR irradiation (0.8 W/cm^2^) for 5 min at a 2.5-h interval. The bacterial suspensions were serially diluted, and the 10^−4^ to 10^−6^ dilutions were inoculated onto Luria–Bertani (LB) culture plates. After overnight incubation at 37 °C, the LB culture plates were imaged, and the CFUs on each plate were counted across all experimental groups. Antibacterial activity was calculated as a percentage of bacterial reduction using the following formula: inhibition rate (IR) (%) = (NC − NS)/NC × 100, where NC and NS represent the average number of colonies in the blank control and sample groups, respectively.

#### Bacterial proliferation assay

Hydrogels from each group were immersed in 3 ml of LB liquid culture medium, with PBS added to the B.C. group and targeted antibiotics added to the P.C. group. Then, 10 μl of *E. coli* or MRSA bacterial suspension (1 × 10^6^ CFU/ml) was added to this liquid medium. All samples were incubated in a shaker at 37 °C for 24 h. At predetermined time intervals, 200 μl of the bacterial suspension was transferred to a sterile 96-well plate, and the absorbance at 600 nm was measured using a microplate reader (NanoDrop, Thermo Fisher, USA).

#### Live/dead bacterial staining assay

The different hydrogels were placed in different wells of a 12-well plate. PBS was added to the B.C. group, and bacteria-specific antibiotics were added to the P.C. group. Each well was incubated with 3 ml of liquid medium containing 60 μl of an *E. coli* or MRSA bacterial suspension (5.0 × 10^9^ CFU/ml). After 24 h of incubation, the samples from each group were washed thrice with PBS and then stained with NucGreen dye (which stains live bacteria green) and EthD-III dye (which stains dead bacteria red) for 15 min. The stained samples were transferred to slides and covered with coverslips. Bacterial viability was assessed using fluorescence microscopy (Carl Zeiss, Germany), and the images were analyzed using the Image-Pro Plus software (Media Cybernetics, Silver Spring, MD, USA).

### In vivo hemostatic assessment

To evaluate the hemostatic efficacy of the hydrogels, both mouse tail (MouTa) resection and partial liver (MouLi) resection models were established. All animal experiments adhered to the guidelines put forth by the National Research Council’s *Guide for the Care and Use of Laboratory Animals*. Female mice (weight 18 to 22 g) were obtained from the Laboratory Animal Center of Hualan Biological Co. Ltd. (Han, China). The animals were randomly assigned to 6 groups: B.C., positive control (P.C.), SQ, SQF, SQB, and SQFB. The B.C. group received no treatment, while commercially available gauze alone was used to treat the P.C. group. Hydrogels were applied to the wounds of each animal, and both the volume of blood loss and the time required for hemostasis were recorded.

### In vivo application of SQFB for promoting the healing of infected wounds

This study was approved by the Animal Management Committee of the First Affiliated Hospital of Zhengzhou University and adhered to the National Institutes of Health guidelines for the care and use of laboratory animals. Male BALB/c mice (4 weeks old) were procured from the Laboratory Animal Center of Hualan Biological Co., Ltd. (Henan, China). All animals were housed in the Laboratory Animal Center of Henan Province and allowed free access to food and water prior to experimentation.

#### Infected wound models

After anesthesia induction, a full-thickness incision (diameter: 0.8 cm) was made on the backs of all mice, and 100 μl of an MRSA suspension was added to each wound. The mice were randomly divided into 5 groups: blank group (no treatment, B.C.), positive control group (commercial dressing, P.C.), SQF group, (SQ/bFGF hydrogel), SQFB group (SQ/bFGF/BP hydrogel), and SQFB + NIR group (SQ/bFGF/BP hydrogel with 808-nm NIR radiation). The wounds were covered with the hydrogel samples or the commercial dressings 24 h after infection.

#### Wound contraction assessment

Images of the wound site were captured using a digital camera (iPhone 12, Apple, USA) at 0, 3, 6, and 12 d, respectively. The wound area was quantified using the Image-Pro Plus software. Wound shrinkage was calculated as follows: wound closure (%) = (*A*_0_ − *A_t_*)/*A*_0_ × 100, where *A*_0_ represents the initial wound area and *A_t_* denotes the wound area at a specified time point.

#### Histological analysis

On day 12, regenerated skin tissues were harvested and fixed using 4% paraformaldehyde. Following fixation, the tissues were embedded in paraffin and sectioned. Histological observations were conducted using hematoxylin and eosin (H&E) staining, Masson’s trichrome staining, and immunohistochemical staining for Ki-67, interleukin6 (IL-6), interleukin10 (IL-10), α-smooth muscle actin (α-SMA), and CD31 (Table S1). Additionally, individual organs, including the brain, heart, liver, spleen, lungs, and kidneys, were obtained from each group of mice. H&E staining was used to evaluate the in vivo histocompatibility of the treatment materials (hydrogels). Further, fresh whole blood samples were also collected to test for liver function and kidney function in each group.

### High-throughput analysis

Skin tissue samples from the B.C. and SQFB + NIR groups were frozen in liquid nitrogen and subsequently pulverized. Total RNA was extracted from the tissue using the TRIzol reagent (Thermo Fisher Scientific, Inc., Waltham, MA, USA), and its concentration was measured using an ultraviolet spectrophotometer. Digital gene expression tag profiling was conducted by GENOSEQ Co. Ltd. (Wuhan, China). Gene Ontology enrichment analysis and Kyoto Encyclopedia of Genes and Genomes pathway enrichment analysis were performed following standard protocols.

### Statistical analysis

The results of this study are presented as mean ± standard deviation. Differences between 2 groups were analyzed using an independent-samples *t* test, while one-way analysis of variance was employed to assess differences among multiple groups. All statistical analyses were performed using GraphPad Prism 8.0, with statistically significant values denoted as *P < 0.05, **P < 0.01, and ***P < 0.001.

## Results

### Preparation and characterization of SQFB

**Fig. 2.**
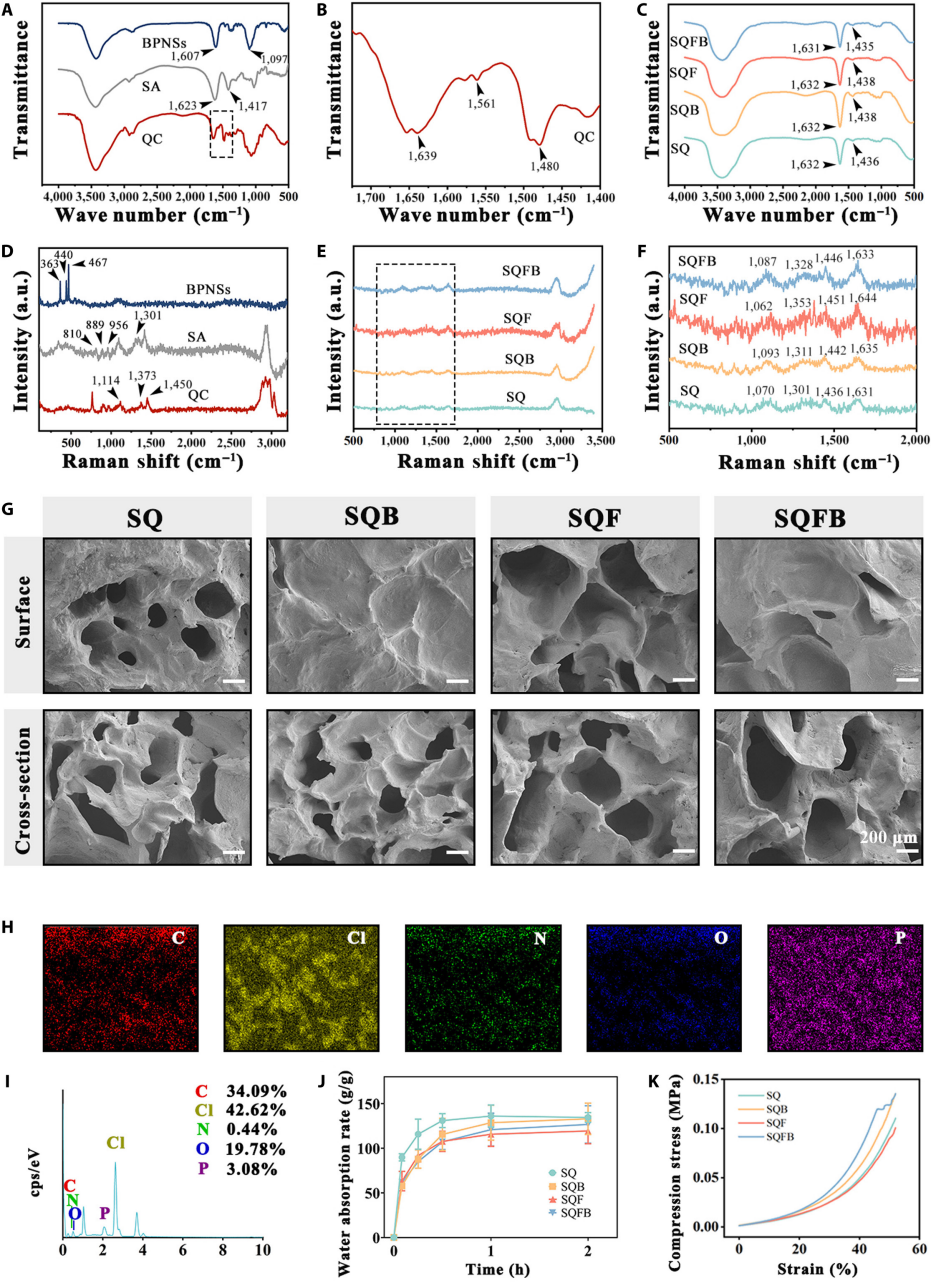
Synthesis and physicochemical evaluation of SQFB. (A to C) Fourier transform infrared spectroscopy (FTIR) spectra of SA, QCS, BPNSs, SQ, SQB, SQF, and SQFB. (D to F) Raman spectra of SA, QCS, BPNSs, SQ, SQB, SQF, and SQFB. (G) Scanning electron microscopy (SEM) images of SQ, SQB, SQF, and SQFB. (H) Energy-dispersive spectroscopy (EDS) surface-scanned elemental mapping images of SQFB. (I) EDS analysis spectrum. (J) Water absorption rate. (K) Typical stress–strain curves showing compressive strength. Scale bars: 40 and 200 μm.

To verify the successful preparation of the hybrid hydrogels, FTIR was performed, and the chemical composition of the hydrogels was characterized. As shown in Fig. 2A, SA exhibited 2 prominent absorption peaks at 1,623 and 1,417 cm^−1^, corresponding to –COO asymmetric and symmetric stretching vibrations, respectively [25]. The 2 broad absorption peaks at 1,097 and 1,607 cm^−1^ arose due to the P–P–O stretching band and P=O vibrational band, respectively [26]. Meanwhile, QCS displayed 3 distinct absorption peaks at 1,639, 1,561, and 1,480 cm^−1^, which corresponded to the C–H stretching vibration of the CH_3_ group in the quaternary ammonium moiety, the C=O stretching vibration of amide I, and the N–H stretching vibration of amide II in the amide moiety, respectively (Fig. 2B). The FTIR spectra of the SQ, SQB, SQF, and SQFB hydrogels showed shifts in peaks corresponding to SA and QCS, as well as altered peak intensities (Fig. 2C).

As shown in Fig. 2D to F, the Raman spectra of the hydrogels were also obtained. The characteristic peaks of SA observed at wavelengths below 1,300 cm^−1^, specifically at 810, 889, and 956 cm^−1^, could be attributed to backbone vibrations (Fig. 2D). Meanwhile, the characteristic peak beyond 1,301 cm^−1^ arose from the stretching vibration of the carboxyl groups in SA [27]. Additionally, BPNSs exhibited characteristic peaks at 363, 440, and 467 cm^−1^, which corresponded to the theoretically calculated absorption peaks of bulk BP (360, 440, and 470 cm^−1^, respectively) [26]. The Raman spectrum of QCS contained 2 characteristic peaks at 1,373 and 1,450 cm^−1^, which corresponded to the stretching vibrations of δ (CH_2_) and δ (CH_3_), respectively [28]. Figure 2E and F shows that the strong electrostatic interactions between the carboxylic anions in SA and the quaternary ammonium cations in QCS significantly increased the peak intensity for the SQ, SQB, SQF, and SQFB groups within the spectral ranges of 750 to 1,750 and 2,750 to 3,250 cm^−1^. Furthermore, the characteristic peaks of SQ were also observed in the other hydrogels, further demonstrating the successful preparation of the various hydrogels.

Subsequently, the morphology and structure of the SQ, SQB, SQF, and SQFB hydrogels were observed using scanning electron microscopy (Fig. 2G). The surface of the SQ hydrogel exhibited uniform pore distribution, but the surface pores became masked after BPNS loading. Furthermore, upon bFGF loading, an enlargement of the pore size on the SQF surface was observed. The cross-section of the SQ, SQB, SQF, and SQFB hydrogels exhibited uniform pores. Here too, the loading of bFGF led to an increase in pore size.

Additionally, the surface of the SQFB hydrogel was characterized using energy-dispersive spectroscopy (EDS) (Fig. 2H and I). Carbon (C, 34.09%), chlorine (Cl, 42.62%), nitrogen (N, 0.44%), oxygen (O, 19.78%), and phosphorus (P, 3.08) were uniformly distributed on the SQFB surface, indicating the successful loading of BPNSs. Nevertheless, these findings were insufficient to confirm bFGF loading, since QCS also contains nitrogen.

As shown in Fig. 2J, when the dry SQ, SQB, SQF, and SQFB gels were immersed in water, the water absorption rate in each group peaked at 30 min before gradually stabilizing. A good water absorption capacity facilitates the absorption of wound tissue exudate and the cleaning of the wound area.

For compression tests, the compression–strain curves of the hydrogels were recorded, as shown in Fig. 2K. The maximum compressive capacity of the SQFB hydrogel was as high as 0.12 MPa, and deformation reached nearly 45%. The remaining hydrogels had a degree of deformation ranging from 45% to 60%, which demonstrated their excellent mechanical properties (Fig. S1). The mechanical properties were in line with those required for wound dressings [29].

### Photothermal performance and drug release behavior of SQFB

**Fig. 3.**
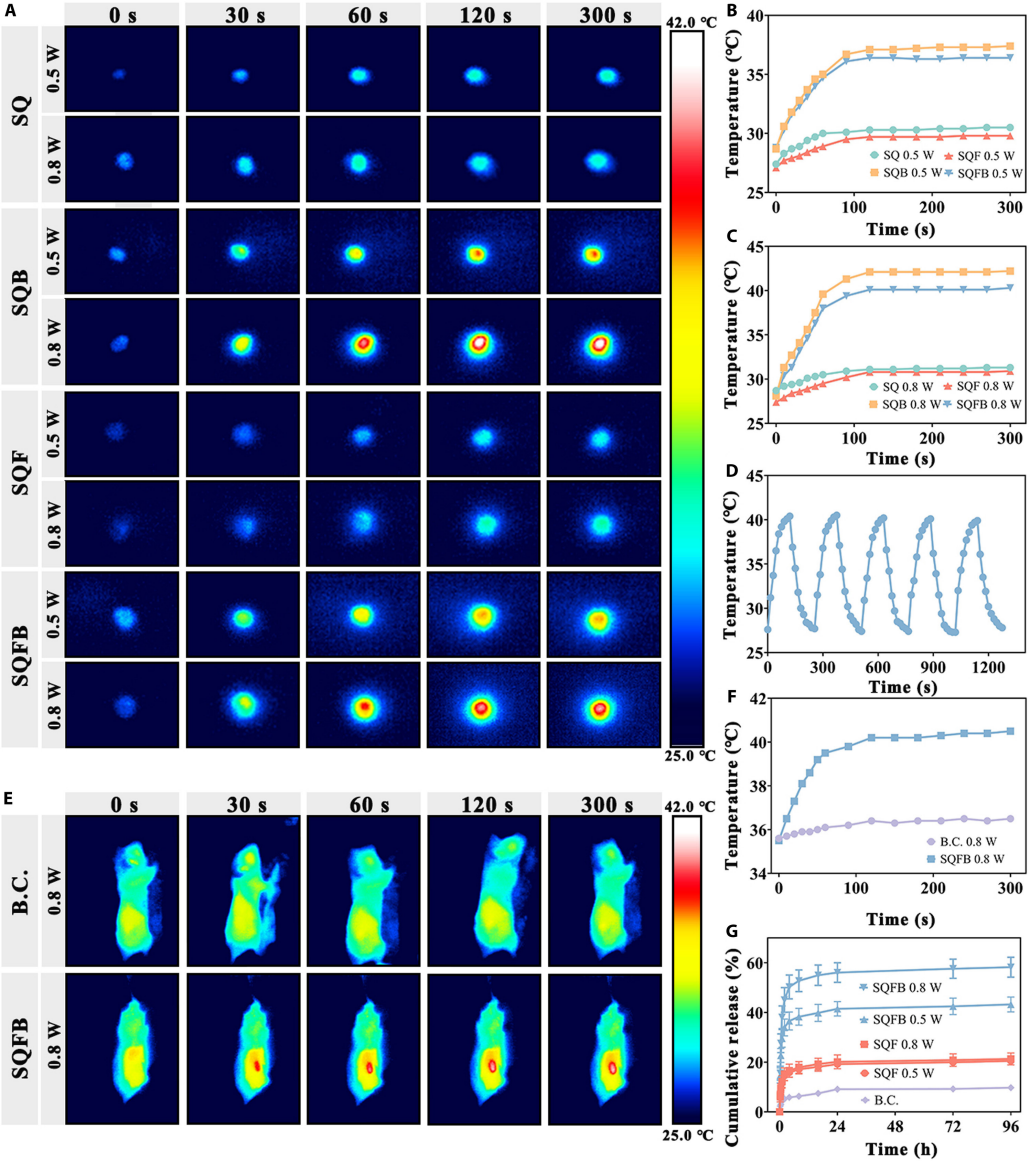
Preparation and physicochemical characterization of SQFB. (A) Representative thermal images of SQ, SQB, SQF, and SQFB exposed to 0.5 and 0.8 W/cm^2^ NIR irradiation for 0, 30, 60, 120, and 300 s. (B and C) Temperature–time curves of the different samples. (D) Temperature profile of SQFB after exposure to 5 NIR laser on/off cycles at 0.8 W/cm^2^. (E) Representative thermal images of the B.C. and SQFB groups of mice subjected to 0.8 W/cm^2^ NIR irradiation for 0, 30, 60, 120, and 300 s. (F) In vivo temperature–time curves in the B.C. and SQFB groups. (G) Cumulative release of bFGF under 0.5 and 0.8 W/cm^2^ NIR irradiation.

Due to the presence of BPNSs, SQFB could generate a significant amount of heat upon exposure to 808-nm NIR irradiation. The thermal images presented in Fig. 3A illustrate the effects of 808-nm NIR irradiation at power densities of 0.5 and 0.8 W/cm^2^ across different groups. In the SQ and SQF gels, which did not contain BPNSs, the temperature increase was minimal regardless of the irradiation power. Under 0.5 W/cm^2^ irradiation, the temperatures of the SQB and SQFB hydrogels rose gradually to 37.1 and 36.4 °C, respectively (Fig. 3B). Under 0.8 W/cm^2^ irradiation, the temperatures of the SQB and SQFB hydrogels increased progressively to 42.1 and 40.1 °C, respectively (Fig. 3C). This indicated that the temperature rise in hydrogels containing BPNSs was faster when the power density of 808-nm NIR irradiation was greater, and the temperature at equilibrium was also higher.

To optimize wound healing and enhance antibacterial effects, 808-nm NIR irradiation at a power density of 0.8 W/cm^2^ was selected for subsequent experiments. The SQFB hydrogel was subjected to NIR exposure for 5 laser on/off cycles to assess the stability of its photothermal conversion performance (Fig. 3D). The body temperature of the B.C. group mice did not change significantly. However, SQFB achieved a temperature of 40.4 °C in about 2 min, and a temperature of 39.9 °C was reached during the fifth cycle. In vivo, the backs of the SQFB group mice reached a temperature of 40.2 °C within 120 s of NIR irradiation (Fig. 3E) before gradually stabilizing (Fig. 3F). In addition, the photothermal conversion efficiency (*η*) of the SQFB hydrogel under NIR irradiation at 0.8 W/cm^2^ was tested. Based on the heat transfer time constant and the maximum steady-state temperature, the *η* value of SQFB was determined to be 41.27% (Fig. S2).

Subsequently, the SQF and SQFB hydrogel samples were immersed in PBS and exposed to 808-nm NIR irradiation (0.5 and 0.8 W/cm^2^) to measure the release of bFGF. The SQFB hydrogel that was not exposed to NIR irradiation was designated as the blank control (B.C.) sample (Fig. 3G). In the B.C. group, only 9.72% ± 0.99% of the total bFGF was released from BPNSs in the absence of NIR irradiation. Meanwhile, the accumulation of bFGF in the SQF group at 96 h was approximately the same following 0.5 and 0.8 W/cm^2^ NIR irradiation (20.50% ± 1.369% and 21.30% ± 2.42%, respectively). However, the accumulation of bFGF at 96 h following 0.5 and 0.8 W/cm^2^ NIR irradiation was higher in the SQFB group (43.21% ± 3.01% and 58.22% ± 4.03%, respectively). The enhanced release of bFGF induced by NIR irradiation is likely attributable to 2 primary factors. Firstly, NIR light irradiation disrupts the interaction between bFGF and BPNSs while simultaneously accelerating the migration of oxygen atoms in the aqueous environment, thereby expediting the degradation of hydrogel components [30]. Secondly, the rise in temperature enhances the mobility of drug molecules, consequently increasing the release of bFGF [31].

The iontophoresis permeation method was employed to examine the transdermal penetration of bFGF across the entire thickness of a pigskin sample (Fig. S3a). Subsequently, ELISA was utilized to measure the cumulative drug concentration in the SQFB groups with and without NIR irradiation (Fig. S3b). The drug release curves revealed that the cumulative release of bFGF at 3 h under NIR irradiation reached 28.70% ± 4.28%. However, in the absence of NIR irradiation, only 8.02% ± 1.27% of the bFGF was released.

### In vitro hemostatic and antibacterial performance

**Fig. 4.**
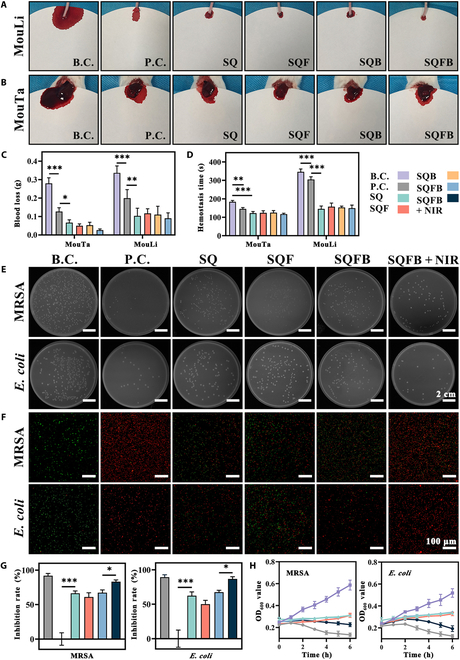
Hemostatic and antibacterial performance of SQFB. (A and B) Representative images of the 2 animal models of hemostasis: mouse tail (MouTa) and partial liver resection (MouLi). (C and D) Quantitative analysis of blood loss and time to hemostasis. (E) Representative images depicting MRSA and *E. coli* colonies on culture plates. (F) Live/dead bacterial staining assays for MRSA and *E. coli*, with live bacteria stained green and dead bacteria stained either red or costained green and red. (G) Quantitative analysis of bacterial colony counts for evaluating bacterial inhibition. (H) Proliferation curves of MRSA and *E. coli*. Scale bars: 2 cm and 100 μm. Significant differences: *P < 0.05, **P < 0.01, and ***P < 0.001.

Rapid hemostasis is important for wound healing. As shown in Fig. 4A and B, we constructed the MouTa and MouLi hemorrhage models to evaluate the hemostatic effect of the different SQ-based hydrogels. In the MouTa model, the blood loss and hemostasis time in the SQ group were reduced to 0.067 ± 0.015 g and 122.33 ± 9.02 s, respectively. A similar trend was also observed in the MouLi model (Fig. 4C and D). There was no significant difference in blood loss and hemostatic time among the SQ, SQF, SQB, and SQFB groups.

Additionally, the blood clotting index (BCI) and platelet adsorption tests were employed to further examine the in vitro hemostatic properties of the hydrogels (Fig. S4). The BCI serves as an indicator of the hydrogel’s in vitro hemostatic efficacy, with lower BCI values indicating a superior hemostatic effect. Meanwhile, the higher the platelet adsorption capacity is, the more rapid is the formation of hemostatic clots at wound sites. Compared to the P.C. group, all 4 hydrogel groups exhibited significantly lower BCI values and significantly higher platelet adsorption rates. This indicated that the hemostatic performance of the SQ hydrogel was not altered by the addition of BP and bFGF. The hemostatic efficacy of the SQFB hydrogel can primarily be attributed to 2 mechanisms: (a) QCS adheres to the bleeding site, thereby initiating the coagulation cascade via the extrinsic coagulation pathway. (b) The electrostatic interactions between QCS and negatively charged erythrocytes facilitate the clotting process at the hemorrhage site [32]. In contrast, the traditional gauze dressing relied solely on filling and compression for hemostasis.

Antibacterial effects are crucial for accelerating the healing of infected wounds. In this study, MRSA and *E. coli* were selected as representative G+ and G− bacteria, respectively, to validate the antibacterial efficacy of the hydrogels (Fig. 4E). While the B.C. culture plates were filled with bacteria (Fig. 4G), the SQ, SQF, and SQFB groups achieved MRSA and *E. coli* IRs of 66.46% ± 3.33% and 62.46% ± 5.45%, 61.06% ± 6.09% and 50.15% ± 5.47%, and 67.27% ± 4.00% and 67.51% ± 2.78%, respectively. Thus, the SQ, SQF, and SQFB hydrogels significantly inhibited bacterial survival. After NIR irradiation, the SQFB + NIR group showed even higher MRSA and *E. coli* IRs than the other experimental groups (83.27% ± 2.57% and 86.80% ± 3.13%, respectively). Figure 4F shows the results of live/dead bacterial staining. Notably, a large number of red-stained dead bacteria were observed in all SQ-based hydrogel groups. Among these groups, the highest number of dead bacteria was observed in the SQFB + NIR group (Fig. S5).

The bacterial proliferation curves for MRSA and *E. coli* (Fig. 4H) indicated that the bacterial OD_600_ values were reduced in all experimental groups. Notably, the SQFB + NIR group displayed a reduction comparable to that of the P.C. group, with the final OD_600_ value being lower than the initial OD_600_ value. SQFB + NIR exhibits excellent antibacterial properties, primarily attributed to the following mechanisms: (a) The positively charged QCS interacts with the negatively charged groups on the bacterial cell surface, altering the fluidity and permeability of the cell membrane, leading to nutrient leakage and exerting antibacterial effects. (b) The abundance of active amino groups in QCS molecules facilitates their adsorption onto bacterial cell membranes, causing flocculation and polymerization, thereby inhibiting bacterial reproduction [33]. (c) The sharp edge of BP can inflict physical damage on the bacterial membrane during interactions with bacteria, leading to RNA leakage and ultimately resulting in bacterial mortality [34]. (d) NIR radiation generates heat, enhancing the permeability of the bacterial cell membrane and consequently accelerating bacterial death [35].

### In vitro cell proliferation and angiogenic effects

**Fig. 5.**
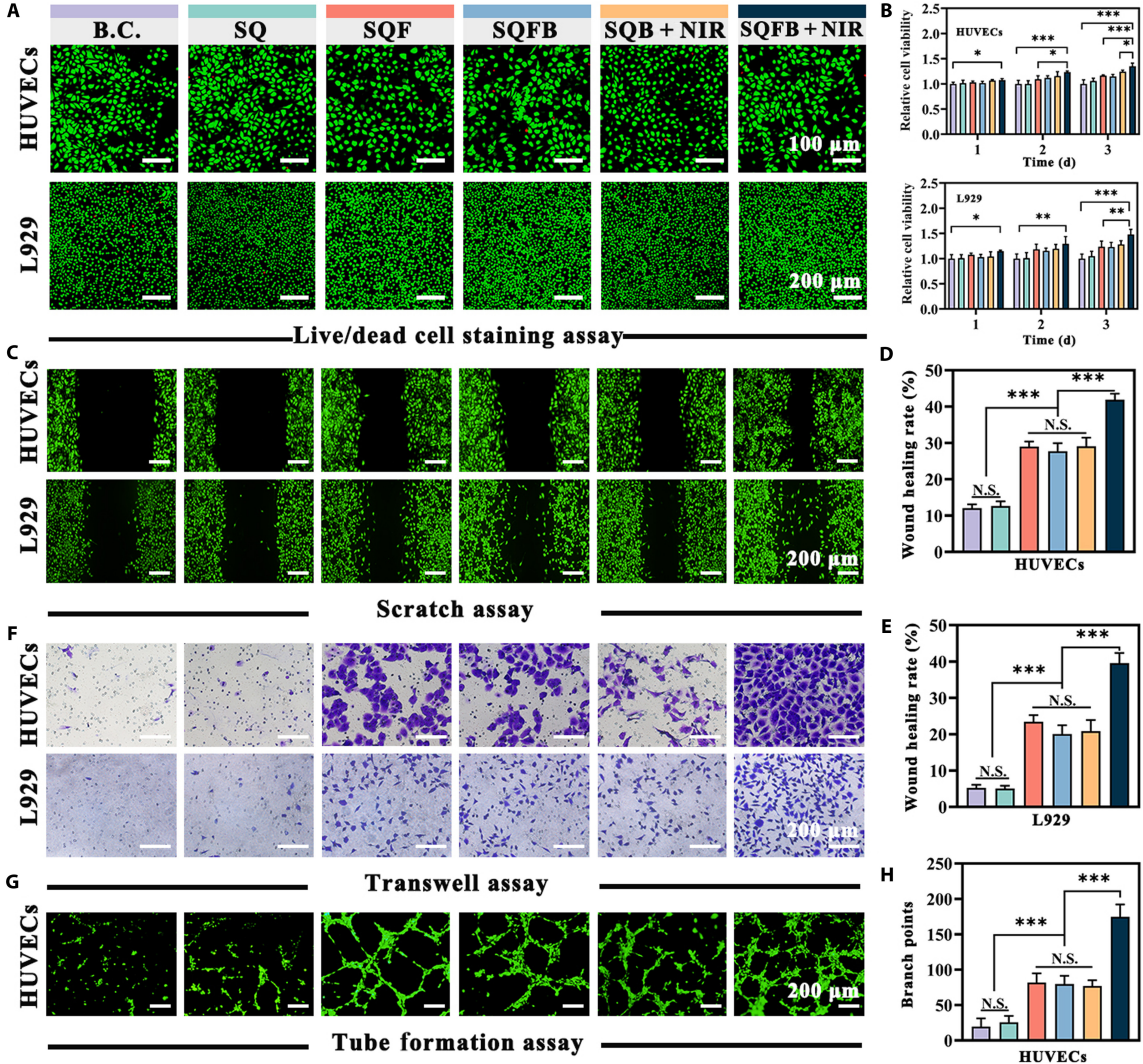
In vitro effect of SQFB on cell proliferation and angiogenesis. (A) Representative images of the live/dead cell staining assay of HUVECs, with green indicating live cells and red indicating dead cells. (B) Quantitative analysis of cell viability in HUVECs and L929 cells. (C) Representative images of calcein-AM staining following the scratch assay. (D and E) Quantitative assessment of the wound healing rates in HUVECs and L929 cells. (F) Representative images from the Transwell assay. (G) Representative images showing calcein-AM staining following the tube formation assay. (H) Quantitative analysis of branch points. Scale bars: 100 and 200 μm. Significant differences: *P < 0.05, **P < 0.01, and ***P < 0.001. N.S., not significant.

This study subsequently evaluated the biocompatibility of SQFB and its effect on promoting cell proliferation and angiogenesis. To this end, mouse fibroblasts (L929 cells) and HUVECs were selected for in vitro assays. As demonstrated in Fig. 5A, the number of red-stained cells in either cell line was minimal across all experimental groups and was comparable to that in the B.C. group. During the live/dead cell staining analysis of HUVECs and L929 cells, the percentage of live cells in the SQFB + NIR group (Fig. S6) was found to be 99.30% ± 0.40% and 99.84% ± 0.15%, respectively. These values were comparable to those in the B.C. group, indicating that the SQFB hydrogel exhibits excellent biocompatibility.

The effect of the SQFB hydrogel on cell proliferation was evaluated using the CCK-8 assay (Fig. 5B). On the first day, the HUVECs and L929 cells in the SQFB + NIR group showed a slightly increased rate of cell proliferation, while no such increase was observed in the other groups. On the second day, in addition to the SQB + NIR group, an increase in cell proliferation was also observed in the SQF and SQFB groups. By day 3, all groups except the SQ group showed an elevated rate of cell proliferation. Notably, the SQFB + NIR group demonstrated a significantly superior cell proliferation performance when compared to the other groups.

As shown in Fig. 5C, the HUVECs and L929 cells were also subjected to scratch assays after treatment with different hydrogels for 24 h. The largest cell-free gaps among HUVECs and L929 cells were observed in the B.C. and SQ groups (12.03% ± 1.07% and 5.24% ± 0.83%, and 12.65% ± 1.26% and 5.05% ± 0.77%, respectively) (Fig. 5D and E). In comparison, the SQFB + NIR group exhibited the most potent scratch healing effect (41.88% ± 1.65% and 39.57% ± 2.78%). As shown in Fig. 5F, a Transwell assay was subsequently conducted to evaluate the effect of the SQFB hydrogel on cell migration. Again, the lowest number of migratory cells was observed in the B.C. and SQ groups (Fig. S7). Some migratory cells were detected in the SQF, SQFB, and SQB + NIR groups, but the highest number of migratory cells was noted in the SQFB + NIR group (216 ± 22.00 and 391 ± 25.24, respectively). Meanwhile, in the HUVEC tube formation assay (Fig. 5G), the B.C. and SQ groups contained almost no mature or complete tube structures. In contrast, mature tube formation could be observed in the SQF, SQFB, and SQB + NIR groups. Among them (Fig. 5H and Fig. S8), the SQFB + NIR group contained the highest number of branch points and capillary length (174.80 ± 17.40 and 138.49 ± 11.68 mm, respectively). Collectively, the findings demonstrated that SQFB + NIR treatment was highly effective at promoting cell proliferation and angiogenesis. The promotion of cell proliferation and angiogenesis by SQFB hydrogel may be attributed to the following factors: (a) Owing to the excellent biocompatibility of QCS, it provides an optimal adhesive matrix for cells and modulates the release of relevant factors, thereby facilitating cell proliferation and angiogenesis [36]. (b) SA has the potential to enhance cell proliferation under specific conditions [37]. (c) The photothermal effect of BPNSs under NIR facilitates the proliferation and migration of endothelial cells, thereby promoting neovascularization [38]. (d) The released bFGF binds to the bFGF receptor, thereby promoting the migration and proliferation of multiple cell types [39].

### Skin reepithelialization and collagen deposition

**Fig. 6.**
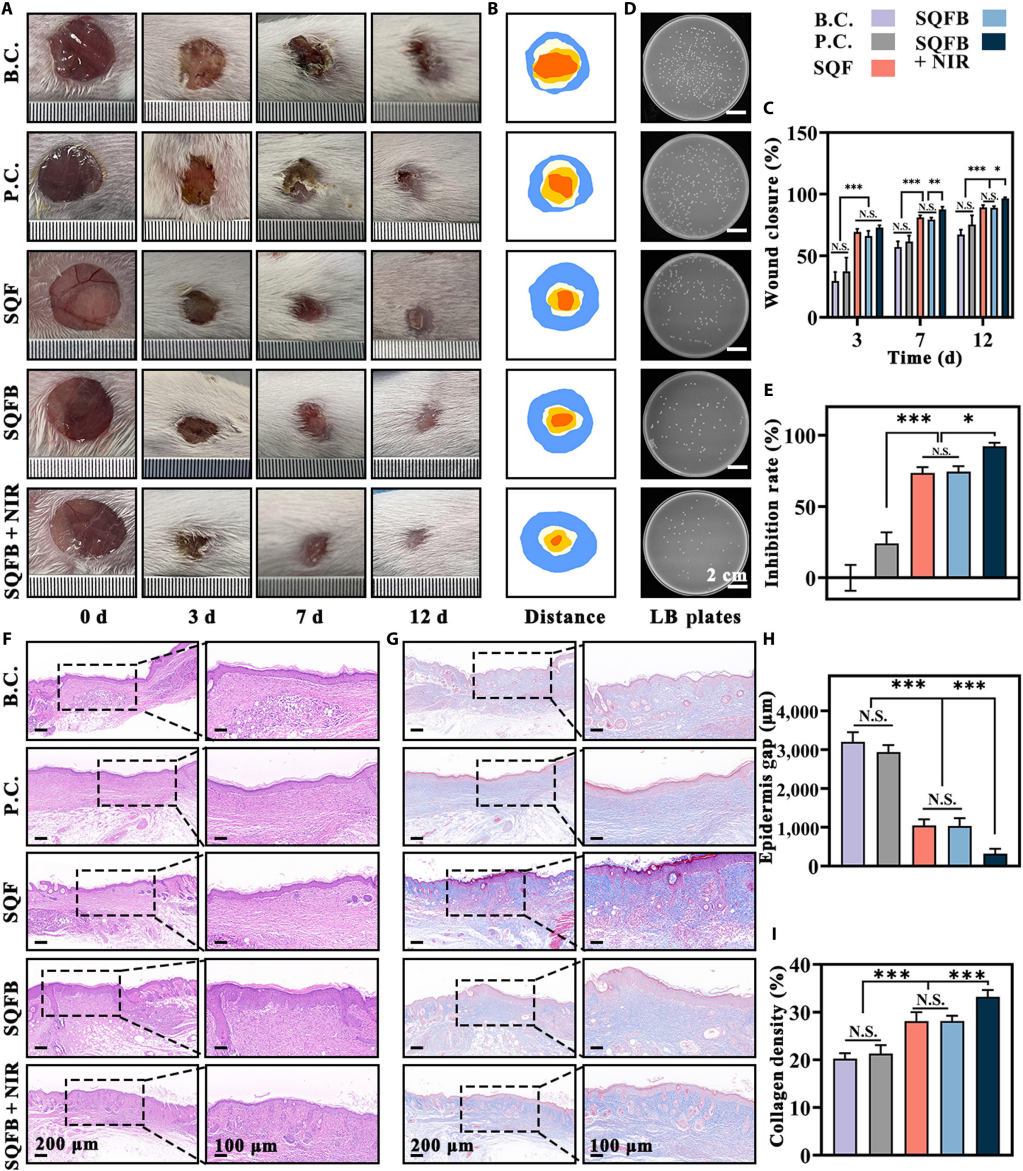
Skin reepithelialization and collagen deposition induced by SQFB + NIR. (A) Images depicting the wound site. (B) Contour map illustrating the wound healing process. (C) Quantitative analysis of wound closure. (D) Number of bacterial colonies isolated from infected wounds (Luria–Bertani [LB] plates). (E) Quantitative analysis of inhibition rates. (F) Representative images of hematoxylin and eosin (H&E) staining at the wound site. (G) Representative images of Masson staining at the wound site. (H) Quantitative analysis of the epidermis gap. (I) Quantitative analysis of collagen density. Scale bars: 100 μm, 200 μm, and 2 cm. Significant differences: *P < 0.05, **P < 0.01, and ***P < 0.001.

A digital camera was used to obtain images, which were subsequently used for the macroscopic reconstruction of mouse wounds. The wounds in the B.C., P.C., SQF, SQFB, and SQFB + NIR groups on day 0, day 3, day 7, and day 12 are presented in Fig. 6A and B. The wounds significantly decreased in size across all groups by day 12. However, the healing rate was significantly lower in the B.C. and P.C. groups than in the SQF, SQFB, and SQFB + NIR groups, reflecting the healing trends observed in infected wounds in clinical settings. Using contour maps to analyze the dynamic wound healing process, wound closure rates at various time points were calculated (Fig. 6C). The SQF, SQFB, and SQFB + NIR groups demonstrated excellent wound healing efficiency on days 3, 7, and 12, outperforming the B.C. and P.C. groups. On day 12, the wound closure rates in the B.C., P.C., and SQFB + NIR groups were 67.28% ± 3.94%, 75.28% ± 7.45%, and 96.58% ± 0.87%, respectively. Notably, by day 12, the SQFB + NIR group exhibited a significantly higher wound closure rate than both the SQF and SQFB groups. Additionally, when bacteria from the wounds were cultured on LB plates (Fig. 6D and E), the number of colonies in the SQFB + NIR group was markedly lower than that in the other groups. This finding indicated that the SQFB + NIR treatment exerted a strong antibacterial effect during the wound healing process.

Epithelial healing is essential for protecting the skin from recurrent invasion by external pathogens. The dermis is primarily composed of collagen, which accounts for more than 90% of its structure. Furthermore, the dermis also contains numerous skin appendages. Notably, collagen deposition is crucial for maintaining skin function [40]. As illustrated in Fig. 6F and G, skin samples were harvested from each group on day 12 for H&E and Masson staining, enabling the assessment of epithelial healing and collagen deposition. In the SQFB + NIR group, there was almost no observable gap in the epidermis, and more intact hair follicles and other skin appendage structures were present. In contrast, the other groups exhibited basic epidermal and dermal structures only. The epidermal gaps in the B.C., P.C., SQF, SQFB, and SQFB + NIR groups were 3,201.60 ± 248.46, 2,938.20 ± 180.01, 1,047.76 ± 156.27, 1,033.82 ± 200.73, and 322.36 ± 122.32 μm, respectively (Fig. 6H). The SQFB + NIR group also displayed a more aligned and coarser deposition of collagen tissue compared to the other groups, and the collagen densities in the B.C., P.C., SQF, SQFB, and SQFB + NIR groups were 20.23% ± 1.16%, 23.11% ± 1.78%, 28.13% ± 1.86%, 28.17% ± 1.08%, and 33.20% ± 1.42%, respectively. Furthermore, the concentrations of bFGF at the wound site in the SQFB + NIR group at 1, 3, and 7 d were significantly higher than those observed in the other SQF and SQFB groups (Fig. S9). Hence, mice receiving SQFB + NIR treatment exhibited the most rapid wound healing rate as well as improved dermal regeneration, collagen density, and skin appendage structures that were comparable to those of normal skin tissue.

### Anti-inflammatory, angiogenic, and scar-tissue-reducing effects

**Fig. 7.**
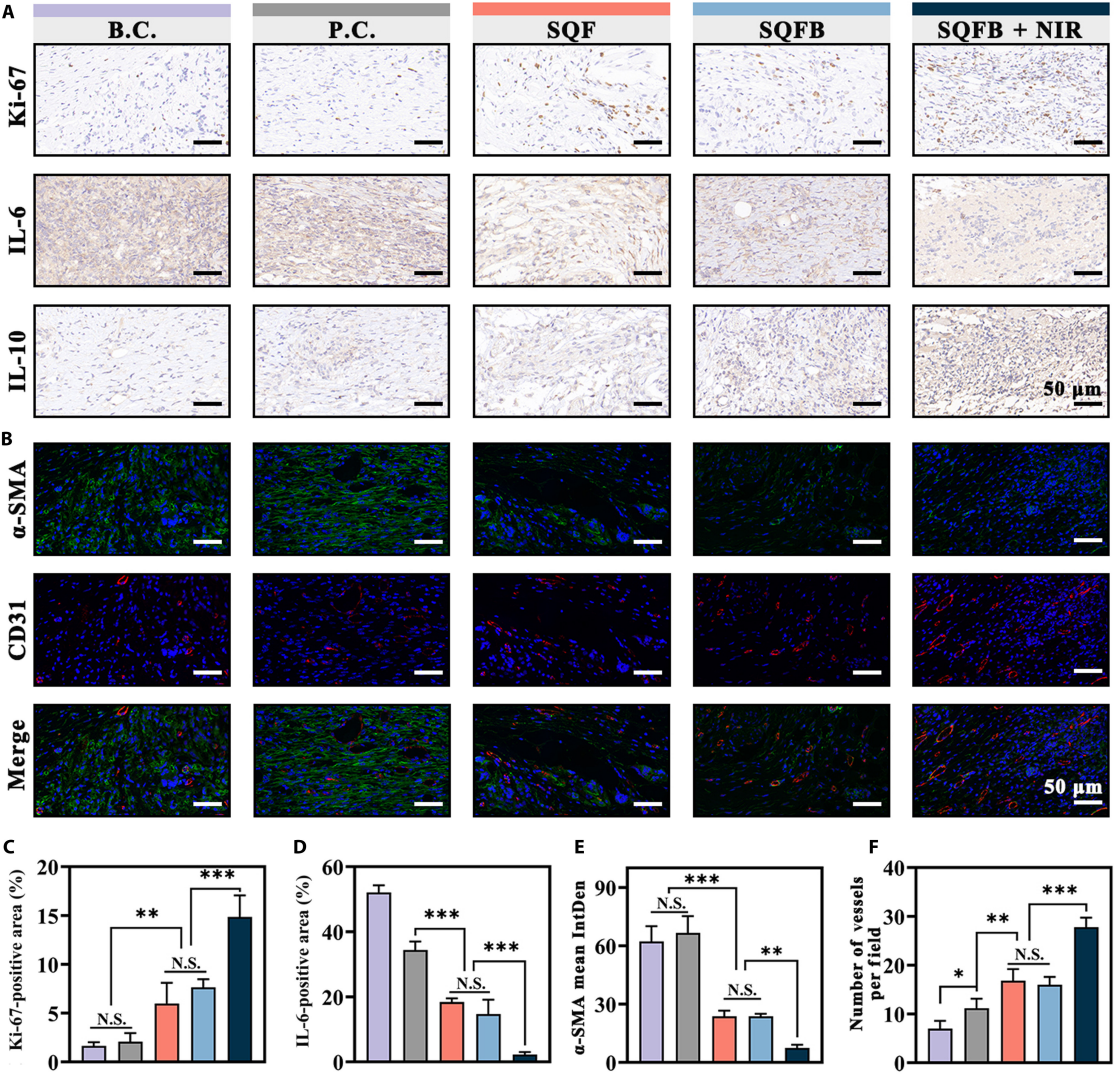
Anti-inflammatory, angiogenic, and scar-tissue-reducing effects of SQFB + NIR. (A) Immunohistochemistry for Ki-67, IL-6, and IL-10 in different groups on day 12. (B) Immunofluorescence for α-SMA/CD31 on day 12. (C) Quantitative analysis of the Ki-67-positive aera. (D) Quantitative analysis of the IL-6-positive aera. (E) Quantitative analysis of the α-SMA mean integrated density (IntDen). (F) Quantitative analysis of the number of vessels per field. Scale bar: 50 μm. Significant differences: *P < 0.05, **P < 0.01, and ***P < 0.001.

Cell proliferation is fundamental to the wound healing process. To assess cell proliferation during wound healing, the expression of the cell-cycle-related marker Ki-67 was evaluated in this study [41]. As shown in Fig. 7A, the immunohistochemical analysis of Ki-67 revealed a significantly larger area of positive Ki-67 expression in wounds from the SQFB + NIR group mice than in wounds from mice belonging to the other groups, consistent with previous in vitro findings of enhanced cell proliferation. The pro-inflammatory factor IL-6 is known to be a marker of chronic wounds and can delay the healing process, while IL-10 acts as an anti-inflammatory factor that maintains the immune balance and promotes epithelial repair [42]. To investigate the anti-inflammatory effects of hydrogel treatment, IL-6 and IL-10 levels were assessed in the different groups using immunohistochemistry. The IL-6-positive area in the B.C., P.C., SQF, SQFB, and SQFB + NIR groups was 52.12% ± 2.15%, 34.41% ± 2.59%, 18.80% ± 1.24%, 13.38% ± 5.35%, and 2.31% ± 0.73%, respectively (Fig. 7D). In contrast, the IL-10-positive area in the B.C., P.C., and SQFB + NIR groups was 2.46% ± 0.93%, 3.08% ± 0.44%, and 31.27% ± 1.86%, respectively (Fig. S10). The trends of IL-10 expression were inversely related to those of IL-6 expression, indicating that the inflammatory response in the SQFB + NIR group was significantly weaker. This reduction in inflammation likely promoted the wound healing process.

Scar tissue represents a suboptimal repair mechanism of wound healing. However, bFGF effectively inhibits the terminal differentiation of myofibroblast cells, thus decreasing the number of α-SMA-positive cells and consequently reducing scar tissue formation [43]. The overexpression of α-SMA by myofibroblasts is strongly associated with scar tissue formation, highlighting its critical role in the fibrotic process. In contrast, CD31, a transmembrane protein, is essential for early angiogenesis, which underscores its importance in vascular development and repair [44]. Thus, immunofluorescence staining for α-SMA and CD31 was employed to evaluate scar tissue and blood vessel formation in wounds in the present study (Fig. 7B). As shown in Fig. 7E, α-SMA expression in the SQFB + NIR group was markedly lower than that in the other groups. Additionally, the number of intact vessels formed by tubular structures expressing CD31 in the B.C., P.C., SQF, SQFB, and SQFB + NIR groups was 7.00 ± 1.58, 11.20 ± 1.92, 16.80 ± 2.39, 16.00 ± 1.58, and 27.80 ± 1.92, respectively (Fig. 7F). These results suggested that bFGF promotes angiogenesis while decreasing the expression of α-SMA-positive cells through its effects on smooth muscle cells and endothelial cells.

The overexpression of transforming growth factor-β1 (TGF-β1) can induce the aberrant proliferation of scar tissue through the production of type I collagen during the process of wound healing. Thus, TGF-β1 expression at the site of wound healing in mice was examined using immunohistochemistry (Fig. S11). The percentage area positive for TGF-β1 in the B.C. and P.C. groups was 17.06% ± 1.78% and 6.60% ± 1.20%, respectively. However, significantly lower percentages were observed in the SQF, SQFB, and SQFB + NIR groups, which were exposed to bFGF release (3.03% ± 0.38%, 2.67% ± 0.84%, and 1.04% ± 0.18%, respectively). In summary, the superior wound healing efficacy of SQFB + NIR treatment could be attributed to 2 main factors: (a) the inherent anti-inflammatory properties of QCS combined with the synergistic photothermal effect induced by BP, leading to an enhanced anti-inflammatory response, and (b) the temperature-responsive hydrogel, which facilitated the sustained release of bFGF under NIR irradiation, promoting more efficient angiogenesis while concurrently inhibiting scar tissue formation.

Biosafety is particularly crucial for the in vivo application of biomaterials. To assess this parameter, fresh whole blood samples were collected and coincubated with the different hydrogel materials for hemolysis experiments. The results showed no significant hemolysis across any of the hydrogel groups (Fig. S12). Subsequently, organ tissue samples—including samples from the heart, liver, spleen, lung, and kidney—were collected for histological analyses. Additionally, fresh plasma was obtained for biochemical assays. H&E staining revealed no discernible differences between the tissues from the experimental and control groups (Fig. S13). Furthermore, the hepatic and renal function indexes of the mice remained within normal ranges across all groups (Fig. S14).

### RNA sequencing analysis of the therapeutic efficacy of SQFB + NIR

**Fig. 8.**
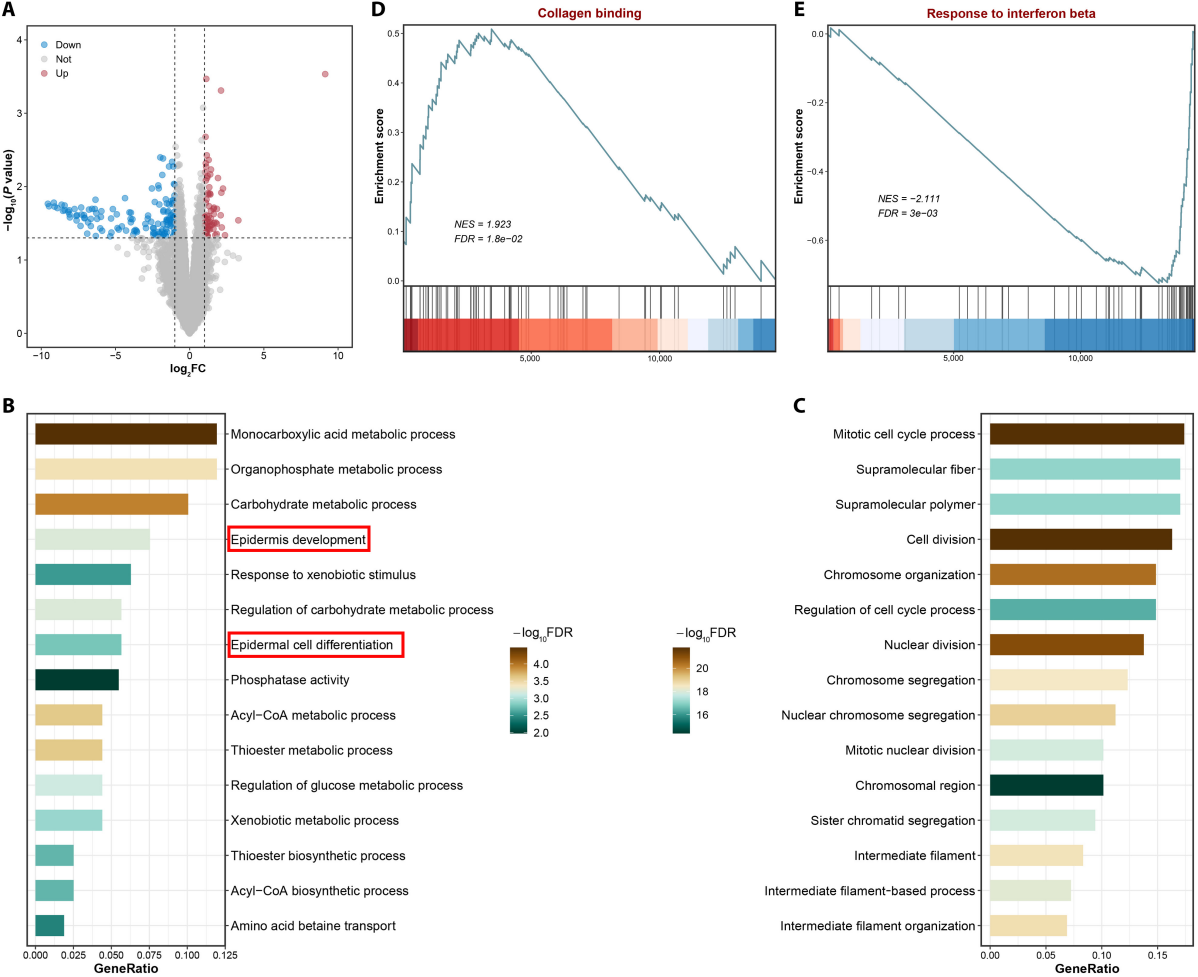
Biological function analysis. (A) Results of edgeR difference analysis between the SQFB + NIR and control groups. (B and C) Gene Ontology (GO) enrichment analysis of genes up-regulated and down-regulated in the SQFB + NIR group. (D and E) Gene set enrichment analysis based on fold changes in gene expression between the SQFB + NIR and control groups. FC, fold change; NES, normalized enrichment score; FDR, false discovery rate.

Following in vitro and in vivo experiments, RNA sequencing was employed to elucidate the molecular mechanisms underlying the healing of infectious wounds following NIR-assisted SQFB treatment. As shown in Fig. 8A, differential analysis yielded 69 genes that were significantly up-regulated and 132 genes that were significantly down-regulated in the experimental group. To further examine the potential biological functions of these differentially expressed genes, enrichment analysis based on Gene Ontology gene sets was performed. The results revealed that the up-regulated genes were enriched for pathways related to epithelial development and metabolism, such as epidermis development and epidermal cell differentiation (Fig. 8B). Conversely, the down-regulated genes were primarily enriched for pathways associated with cell cycle regulation (Fig. 8C). Moreover, gene set enrichment analysis demonstrated a significant up-regulation of collagen binding—which is closely linked to skin repair and regeneration—in the SQFB + NIR group (Fig. 8D). Additionally, a notable down-regulation of the pathways involved in inflammation and response to interferon beta was observed (Fig. 8E). In conclusion, the results indicated that NIR-assisted SQFB treatment promotes the healing of infected wounds by regulating the interaction between epithelial development and metabolism during repair.

## Discussion

Infection is an important factor contributing to the delayed healing of open wounds, such as those resulting from burns, trauma, and surgical procedures. Open wounds are particularly vulnerable to bacterial infections and can develop into infected wounds. If bacteria enter the bloodstream and cause bacteremia, a severe inflammatory response is triggered, leading to the risk of multiple organ failure and life-threatening injuries. Additionally, the formation of bacterial biofilms can transform acute wounds into chronic ones, further complicating the healing process.

In this study, we selected BPNSs and bFGF to synergically enhance the healing of infected wounds. BPNSs exhibit a remarkably high photothermal conversion efficiency owing to their unique 2-dimensional structure [45]. Additionally, QCS has also shown immense potential as a drug carrier for growth factors such as bFGF. While nonphotoresponsive hydrogels allow for the continuous release of bFGF, photoresponsive hydrogels offer several advantages: (a) The BPNSs in the hydrogel exhibit a high surface-to-volume ratio, which enhances the loading capacity and bioavailability of bFGF. (b) NIR-irradiation-induced high temperatures facilitate the collision and movement of bFGF molecules loaded on BPNSs, thereby increasing the drug release rate. (c) Thermal stimulation at optimal temperature levels promotes tissue regeneration to a certain extent.

Importantly, in this study, RNA sequencing analysis revealed that the SQFB + NIR group exhibited a significant up-regulation of pathways associated with epidermal development and epidermal cell differentiation. Previous studies have demonstrated that bFGF activates the PI3K/Akt pathway, thereby stimulating cell proliferation [46]. Moreover, bFGF can inhibit the transformation of epidermal stem cells into myofibroblasts by activating the Notch1/Jagged1 pathway, effectively suppressing scar tissue proliferation [47]. Based on our hypothesis, it is plausible that SQFB + NIR treatment not only stimulates epidermal cell proliferation to provide a larger pool for epidermal development but also enhances wound healing by promoting cell migration. Additionally, the bFGF within the gel facilitates cell differentiation by regulating the expression of key transcription factors [48]. Furthermore, both bFGF and NIR irradiation may induce angiogenesis to supply essential nutrients and oxygen for wound healing and cell differentiation [49,50].

Overall, we have developed a promising QCS-based hybrid hydrogel with intrinsic antibacterial properties as a novel dressing material for the treatment of infected wounds, aiming to enhance wound healing. This hydrogel was engineered to optimize its pore sizes and allow for the efficient storage and controlled release of bFGF in response to photothermal stimulation. This controlled release mechanism not only enhances the therapeutic efficacy of the drug but also establishes a connection between molecular-scale dynamics and macroscopic outcomes. The SQFB hydrogel exhibits exceptional antibacterial activity, promotes cell proliferation and angiogenesis, and possesses hemostatic properties. Our in vivo experiments have demonstrated that this innovative dressing can expedite skin repair and diminish scar tissue formation. Hydrogels enable the seamless integration of biomaterials with photothermal therapies, as well as with photodynamic and chemodynamic therapies. This study represents a significant advancement in wound treatment and has promising clinical implications. However, our use of a rodent model presents certain limitations. Thus, future investigations would benefit from employing primate or humanoid models for greater clinical relevance.

## Data Availability

The datasets supporting the conclusions of this article are included within the article and its Supplementary Materials.

## References

[1] Abe M, Yokoyama Y, Ishikawa O. A possible mechanism of basic fibroblast growth factor-promoted scarless wound healing: The induction of myofibroblast apoptosis. Eur J Dermatol. 2012;22(1):46–53. 22370167 10.1684/ejd.2011.1582

[2] Liu W, Gao R, Yang C, Feng Z, Ou-Yang W, Pan X, Huang P, Zhang C, Kong D, Wang W. ECM-mimetic immunomodulatory hydrogel for methicillin-resistant *Staphylococcus aureus*–infected chronic skin wound healing. Sci Adv. 2022;8(27): Article eabn7006. 35857459 10.1126/sciadv.abn7006 PMC9269894

[3] Kurian AG, Mandakhbayar N, Singh RK, Lee JH, Kim HW. Multifunctional molybdenum-based nanoclusters engineered gelatin methacryloyl as in situ photo-cross-linkable hybrid hydrogel dressings for enhanced wound healing. ACS Appl Mater Interfaces. 2024;16(27):34641–34655. 38934374 10.1021/acsami.4c05636

[4] Kurian AG, Singh RK, Sagar V, Lee JH, Kim HW. Nanozyme-engineered hydrogels for anti-inflammation and skin regeneration. Nanomicro Lett. 2024;16(1): Article 110. 38321242 10.1007/s40820-024-01323-6 PMC10847086

[5] Pelgrift RY, Friedman AJ. Nanotechnology as a therapeutic tool to combat microbial resistance. Adv Drug Deliv Rev. 2013;65(13–14):1803–1815. 23892192 10.1016/j.addr.2013.07.011

[6] Hernández-González AC, Téllez-Jurado L, Rodríguez-Lorenzo LM. Alginate hydrogels for bone tissue engineering, from injectables to bioprinting: A review. Carbohydr Polym. 2020;229: Article 115514. 31826429 10.1016/j.carbpol.2019.115514

[7] Pal K, Bharti D, Sarkar P, Anis A, Kim D, Chalas R, Maksymiuk P, Stachurski P, Jarzebski M. Selected applications of chitosan composites. Int J Mol Sci. 2021;22(20): Article 10968. 34681625 10.3390/ijms222010968 PMC8535947

[8] Liang Y, Li Z, Huang Y, Yu R, Guo B. Dual-dynamic-bond cross-linked antibacterial adhesive hydrogel sealants with on-demand removability for post-wound-closure and infected wound healing. ACS Nano. 2021;15(4):7078–7093. 33764740 10.1021/acsnano.1c00204

[9] Zhao X, Guo B, Wu H, Liang Y, Ma PX. Injectable antibacterial conductive nanocomposite cryogels with rapid shape recovery for noncompressible hemorrhage and wound healing. Nat Commun. 2018;9: Article 2784. 30018305 10.1038/s41467-018-04998-9 PMC6050275

[10] Cecerska-Heryć E, Goszka M, Serwin N, Roszak M, Grygorcewicz B, Heryć R, Dołęgowska B. Applications of the regenerative capacity of platelets in modern medicine. Cytokine Growth Factor Rev. 2022;64:84–94. 34924312 10.1016/j.cytogfr.2021.11.003

[11] Akita S, Akino K, Hirano A. Basic fibroblast growth factor in scarless wound healing. Adv Wound Care. 2013;2(2):44–49. 10.1089/wound.2011.0324 PMC3623580 24527324

[12] Nunes QM, Li Y, Sun CY, Kinnunen TK, Fernig DG. Fibroblast growth factors as tissue repair and regeneration therapeutics. PeerJ. 2016;4: Article e1535. 26793421 10.7717/peerj.1535 PMC4715458

[13] Wang TL, Zhou ZF, Liu JF, Hou XD, Zhou Z, Dai YL, Hou ZY, Chen F, Zheng LP. Donut-like MOFs of copper/nicotinic acid and composite hydrogels with superior bioactivity for rh-bFGF delivering and skin wound healing. J Nanobiotechnology. 2021;19(1): Article 275. 34503490 10.1186/s12951-021-01014-z PMC8427876

[14] Huo J, Jia Q, Huang H, Zhang J, Li P, Dong X, Huang W. Emerging photothermal-derived multimodal synergistic therapy in combating bacterial infections. Chem Soc Rev. 2021;50:8762–8789. 34159993 10.1039/d1cs00074h

[15] Tao B, Lin C, Deng Y, Yuan Z, Shen X, Chen M, He Y, Peng Z, Hu Y, Cai K. Copper-nanoparticle-embedded hydrogel for killing bacteria and promoting wound healing with photothermal therapy. J Mater Chem B. 2019;7:2534–2548. 32255130 10.1039/c8tb03272f

[16] Patel KD, Singh RK, Kim H-W. Carbon-based nanomaterials as an emerging platform for theranostics. Mater Horizons. 2019;6:434–469.

[17] Singh RK, Patel KD, Leong KW, Kim HW. Progress in nanotheranostics based on mesoporous silica nanomaterial platforms. ACS Appl Mater Interfaces. 2017;9:10309–10337. 28274115 10.1021/acsami.6b16505

[18] Cheng S, Qi M, Li W, Sun W, Li M, Lin J, Bai X, Sun Y, Dong B, Wang L. Dual-responsive nanocomposites for synergistic antibacterial therapies facilitating bacteria-infected wound healing. Adv Healthc Mater. 2023;12(6): Article e2202652. 36373219 10.1002/adhm.202202652

[19] Chen W, Ouyang J, Liu H, Chen M, Zeng K, Sheng J, Liu Z, Han Y, Wang L, Li J, et al. Black phosphorus nanosheet-based drug delivery system for synergistic photodynamic/photothermal/chemotherapy of cancer. Adv Mater. 2017;29(5): Article 1603864 10.1002/adma.201603864. 10.1002/adma.201603864 27882622

[20] Gnanasekar S, Kasi G, He X, Zhang K, Xu L, Kang ET. Recent advances in engineered polymeric materials for efficient photodynamic inactivation of bacterial pathogens. Bioact Mater. 2023;21:157–174. 36093325 10.1016/j.bioactmat.2022.08.011 PMC9421094

[21] Ding Q, Sun T, Su W, Jing X, Ye B, Su Y, Zeng L, Qu Y, Yang X, Wu Y, et al. Bioinspired multifunctional black phosphorus hydrogel with antibacterial and antioxidant properties: A stepwise countermeasure for diabetic skin wound healing. Adv Healthc Mater. 2022;11: Article e2102791. 35182097 10.1002/adhm.202102791

[22] Zhang X, Chen G, Liu Y, Sun L, Sun L, Zhao Y. Black phosphorus-loaded separable microneedles as responsive oxygen delivery carriers for wound healing. ACS Nano. 2020;14(5):5901–5908. 32315159 10.1021/acsnano.0c01059

[23] Zhao Y, Tian C, Liu Y, Liu Z, Li J, Wang Z, Han X. All-in-one bioactive properties of photothermal nanofibers for accelerating diabetic wound healing. Biomaterials. 2023;295: Article 122029. 36731368 10.1016/j.biomaterials.2023.122029

[24] Wu S, Liu G, Shao P, Lin X, Yu J, Chen H, Li H, Feng S. Transdermal sustained release properties and anti-photoaging efficacy of liposome-thermosensitive hydrogel system. Adv Healthc Mater. 2024;13(2): Article e2301933. 37607774 10.1002/adhm.202301933

[25] Jin F, Liao S, Li W, Jiang C, Wei Q, Xia X, Wang Q. Amphiphilic sodium alginate-polylysine hydrogel with high antibacterial efficiency in a wide pH range. Carbohydr Polym. 2023;299: Article 120195. 36876766 10.1016/j.carbpol.2022.120195

[26] Tang M, Zhang X, Yang A, Liu Y, Xie K, Zhou Y, Wang C, Liu J, Shi P, Lin X. Injectable black phosphorus nanosheets for wireless nongenetic neural stimulation. Small. 2022;18(8): Article e2105388. 34894073 10.1002/smll.202105388

[27] Jayeoye TJ, Rujiralai T. Green, *in situ* fabrication of silver/poly(3-aminophenyl boronic acid)/sodium alginate nanogel and hydrogen peroxide sensing capacity. Carbohydr Polym. 2020;246: Article 116657. 32747289 10.1016/j.carbpol.2020.116657

[28] Han P, Li Z, Wei X, Tang L, Li M, Liang Z, Yin X, Wei S. Ion-imprinted thermosensitive chitosan derivative for heavy metal remediation. Carbohydr Polym. 2020;248: Article 116732. 32919549 10.1016/j.carbpol.2020.116732

[29] Zhang M, Zhang Q, Chen X, Jiang T, Song P, Wang B, Zhao X. Mussel-inspired nanocomposite hydrogel based on alginate and antimicrobial peptide for infected wound repair. Int J Biol Macromol. 2022;219:1087–1099. 36049562 10.1016/j.ijbiomac.2022.08.165

[30] Zhang F, Peng F, Qin L, Yang D, Li R, Jiang S, He H, Zhang P. pH/near infrared dual-triggered drug delivery system based black phosphorus nanosheets for targeted cancer chemo-photothermal therapy. Colloids Surf B Biointerfaces. 2019;180:353–361. 31077863 10.1016/j.colsurfb.2019.04.021

[31] Liu S, Liu Z, Wu M, Xu X, Huang F, Zhang L, Liu Y, Shuai Q. NIR as a “trigger switch” for rapid phase change, on-demand release, and photothermal synergistic antibacterial treatment with chitosan-based temperature-sensitive hydrogel. Int J Biol Macromol. 2021;191:344–358. 34560148 10.1016/j.ijbiomac.2021.09.093

[32] Zhao X, Wu H, Guo B, Dong R, Qiu Y, Ma PX. Antibacterial anti-oxidant electroactive injectable hydrogel as self-healing wound dressing with hemostasis and adhesiveness for cutaneous wound healing. Biomaterials. 2017;122:34–47. 28107663 10.1016/j.biomaterials.2017.01.011

[33] Rashki S, Asgarpour K, Tarrahimofrad H, Hashemipour M, Ebrahimi MS, Fathizadeh H, Khorshidi A, Khan H, Marzhoseyni Z, Salavati-Niasari M, et al. Chitosan-based nanoparticles against bacterial infections. Carbohydr Polym. 2021;251: Article 117108. 33142645 10.1016/j.carbpol.2020.117108

[34] Guo T, Zhuang SH, Qiu HL, Guo YT, Wang LL, Jin GX, Lin WW, Huang GM, Yang HH. Black phosphorus nanosheets for killing bacteria through nanoknife effect. Part Part Syst Charact. 2020;37(8): Article 2000169.

[35] Maleki A, He J, Bochani S, Nosrati V, Shahbazi MA, Guo B. Multifunctional photoactive hydrogels for wound healing acceleration. ACS Nano. 2021;15(12):18895–18930. 34870413 10.1021/acsnano.1c08334

[36] Busilacchi A, Gigante A, Mattioli-Belmonte M, Manzotti S, Muzzarelli RA. Chitosan stabilizes platelet growth factors and modulates stem cell differentiation toward tissue regeneration. Carbohydr Polym. 2013;98(1):665–676. 23987397 10.1016/j.carbpol.2013.06.044

[37] Del Gaudio P, Amante C, Civale R, Bizzarro V, Petrella A, Pepe G, Campiglia P, Russo P, Aquino RP. In situ gelling alginate-pectin blend particles loaded with Ac2-26: A new weapon to improve wound care armamentarium. Carbohydr Polym. 2020;227: Article 115305. 31590879 10.1016/j.carbpol.2019.115305

[38] Zhao YN, Liu YM, Tian C, Liu ZQ, Wu KP, Zhang CZ, Han XW. Construction of antibacterial photothermal PCL/AgNPs/BP nanofibers for infected wound healing. Mater Des. 2023;226: Article 111670.

[39] Li X, Wang C, Xiao J, McKeehan WL, Wang F. Fibroblast growth factors, old kids on the new block. Semin Cell Dev Biol. 2016;53:155–167. 26768548 10.1016/j.semcdb.2015.12.014 PMC4875805

[40] Huang J, Heng S, Zhang W, Liu Y, Xia T, Ji C, Zhang LJ. Dermal extracellular matrix molecules in skin development, homeostasis, wound regeneration and diseases. Semin Cell Dev Biol. 2022;128:137–144. 35339360 10.1016/j.semcdb.2022.02.027

[41] Pereira Beserra F, Sérgio Gushiken LF, Vieira AJ, Augusto Bérgamo D, Luísa Bérgamo P, Oliveira de Souza M, Alberto Hussni C, Kiomi Takahira R, Henrique Nóbrega R, Monteiro Martinez ER, et al. From inflammation to cutaneous repair: Topical application of lupeol improves skin wound healing in rats by modulating the cytokine levels, NF-κB, Ki-67, growth factor expression, and distribution of collagen fibers. Int J Mol Sci. 2020;21(14): Article 4952. 32668794 10.3390/ijms21144952 PMC7404060

[42] Brown MS, Ashley B, Koh A. Wearable technology for chronic wound monitoring: Current dressings, advancements, and future prospects. Front Bioeng Biotechnol. 2018;6: Article 47. 10.3389/fbioe.2018.00047 PMC5932176 29755977

[43] Tiede S, Ernst N, Bayat A, Paus R, Tronnier V, Zechel C. Basic fibroblast growth factor: A potential new therapeutic tool for the treatment of hypertrophic and keloid scars. Ann Anat. 2009;191(1):33–44. 19071002 10.1016/j.aanat.2008.10.001

[44] Geng Z, Yu Y, Li Z, Ma L, Zhu S, Liang Y, Cui Z, Wang J, Yang X, Liu C. miR-21 promotes osseointegration and mineralization through enhancing both osteogenic and osteoclastic expression. Mater Sci Eng C Mater Biol Appl. 2020;111: Article 110785. 32279740 10.1016/j.msec.2020.110785

[45] Tao W, Zhu X, Yu X, Zeng X, Xiao Q, Zhang X, Ji X, Wang X, Shi J, Zhang H, et al. Black phosphorus nanosheets as a robust delivery platform for cancer theranostics. Adv Mater. 2017;29(1): Article 1603276 10.1002/adma.201603276. 10.1002/adma.201603276 PMC5205548 27797119

[46] Prudovsky I. Cellular mechanisms of FGF-stimulated tissue repair. Cells. 2021;10(7): Article 1830. 34360000 10.3390/cells10071830 PMC8304273

[47] Wang P, Shu B, Xu Y, Zhu J, Liu J, Zhou Z, Chen L, Zhao J, Liu X, Qi S, et al. Basic fibroblast growth factor reduces scar by inhibiting the differentiation of epidermal stem cells to myofibroblasts via the Notch1/Jagged1 pathway. Stem Cell Res Ther. 2017;8: Article 114. 28511663 10.1186/s13287-017-0549-7 PMC5434520

[48] Huang F, Gao T, Wang W, Wang L, Xie Y, Tai C, Liu S, Cui Y, Wang B. Engineered basic fibroblast growth factor-overexpressing human umbilical cord-derived mesenchymal stem cells improve the proliferation and neuronal differentiation of endogenous neural stem cells and functional recovery of spinal cord injury by activating the PI3K-Akt-GSK-3β signaling pathway. Stem Cell Res Ther. 2021;12(1): Article 468. 34419172 10.1186/s13287-021-02537-w PMC8379754

[49] Montero RB, Vial X, Nguyen DT, Farhand S, Reardon M, Pham SM, Tsechpenakis G, Andreopoulos FM. bFGF-containing electrospun gelatin scaffolds with controlled nano-architectural features for directed angiogenesis. Acta Biomater. 2012;8(5):1778–1791. 22200610 10.1016/j.actbio.2011.12.008 PMC3432918

[50] Zhu S, Zhao B, Li M, Wang H, Zhu J, Li Q, Gao H, Feng Q, Cao X. Microenvironment responsive nanocomposite hydrogel with NIR photothermal therapy, vascularization and anti-inflammation for diabetic infected wound healing. Bioact Mater. 2023;26:306–320. 36950149 10.1016/j.bioactmat.2023.03.005 PMC10027510

