# Supplementary material for: Photothermally Responsive Hydrogel Releases Basic Fibroblast Growth Factor to Promote the Healing of Infected Wounds
Source: Biomater Res. 2025 Mar 4;29:0156. doi: 10.34133/bmr.0156 (PMC11876544; doi:10.34133/bmr.0156)
Supplement: Supplementary 1 — Supplementary Text Table S1 Figs. S1 to S14 [file bmr.0156.f1.docx]

**Supporting Information**

**Experimental Details**

**Photothermal conversion efficiency**

The photothermal conversion efficiency (η) was calculated by the formulae:

η= (hS(Tmax-Tsurr)-Qdis)/I(1-10-A), hS = ΣmCp/τS, τS = - t/lnθ, θ = (T - Tsurr)/(Tmax - Tsurr), where h is the heat transfer coefficient, S is the surface area of the vessel, τS is the time constant of heat transfer in the system, m is the mass of the product, Cp is the specific heat capacity of the solvent, Tmax is the equilibriuxm temperature of the SQFB, Tsurr is the ambient temperature, I is the laser power density, and A is the absorbance of the SQFB at 808 nm.

**Blood clotting index**

The blood clotting index (BCI) is an indicator used to characterize the coagulation behavior of a material. Cylindrical hydrogel samples with a diameter of 6 mm and height of 4 mm were prepared and placed in 1.5 ml EP tubes. Subsequently, 200 µL of fresh rabbit whole blood (0.01 M CaCl_2_, 3.2% sodium citrate anticoagulant) was gradually dripped onto the surface of the hydrogel to ensure complete coverage. The samples were then incubated at 37 °C for 3 min, followed by a specified duration of washing with 5 mL of deionized water to remove any uncoagulated blood. A reference sample was prepared by diluting 200 µL of blood with 5 mL of deionized water and measuring the OD value at 540 nm. BCI (%) = A_sample_/A_reference_ ×100%.

**Platelet adsorption assays**

For platelet adsorption assays, cylindrical hydrogel samples measuring 4 mm in diameter and 2 mm in height were prepared and placed in EP tubes. Subsequently, 100 µL of platelet-rich plasma was gradually dripped onto the surface of the hydrogel to ensure complete coverage. The samples were then incubated at 37 °C for a duration of 10 min. Following this, the samples underwent three washes with PBS before being lysed in 0.5 mL of 1% Triton X-100 and further incubated for 1 h at 37 °C. OD values were measured at a wavelength of 490 nm using a reference sample consisting of 100 µL of platelet-rich plasma. Attachment of platelets (%) = A_sample_/A_reference_ × 100%.

**Concentrations of bFGF at the wound site**

The infected wound mouse models were constructed following established protocols. SQF and SQFB hydrogels were applied to the wounds, with or without NIR irradiation. At 1d, 3d, and 7d post-treatment, the wound bed and dorsal skin surrounding the wound were incised and cut into pieces in a PBS solution (500 µL). The resulting mixture was transferred to a centrifuge tube and centrifuged at 300 g for 5 min. The supernatant was collected and stored at -80 °C for further analysis. bFGF levels were determined using an ELISA kit (Wuhan Fine Biotech Co., Ltd.) according to the manufacturer's instructions. The concentrations of bFGF were calculated by correlating them with a standard curve.

**Table S1**

| Antigens | Species antibodies raised in | Dilution  (IF) | Dilution  (IHC) | Dilution  (WB) | Supplier |
| --- | --- | --- | --- | --- | --- |
| Ki67 | Rabbit, monoclonal | - | 1:500 | - | Wuhan servicebio technology, CHN, Cat. GB111141 |
| IL-6 | Rabbit, polyclonal | - | 1:200 | - | Wuhan servicebio technology, CHN, Cat. GB11117 |
| IL-10 | Rabbit, polyclonal | - | 1:200 | - | Wuhan servicebio technology, CHN, Cat. GB11108 |
| α-SMA | Mouse, monoclonal | 1:200 | - | - | Wuhan servicebio technology, CHN, Cat. #GB13044 |
| CD31 | Rabbit, polyclonal | 1:200 | - | - | Wuhan servicebio technology, CHN, Cat. GB11063-2 |


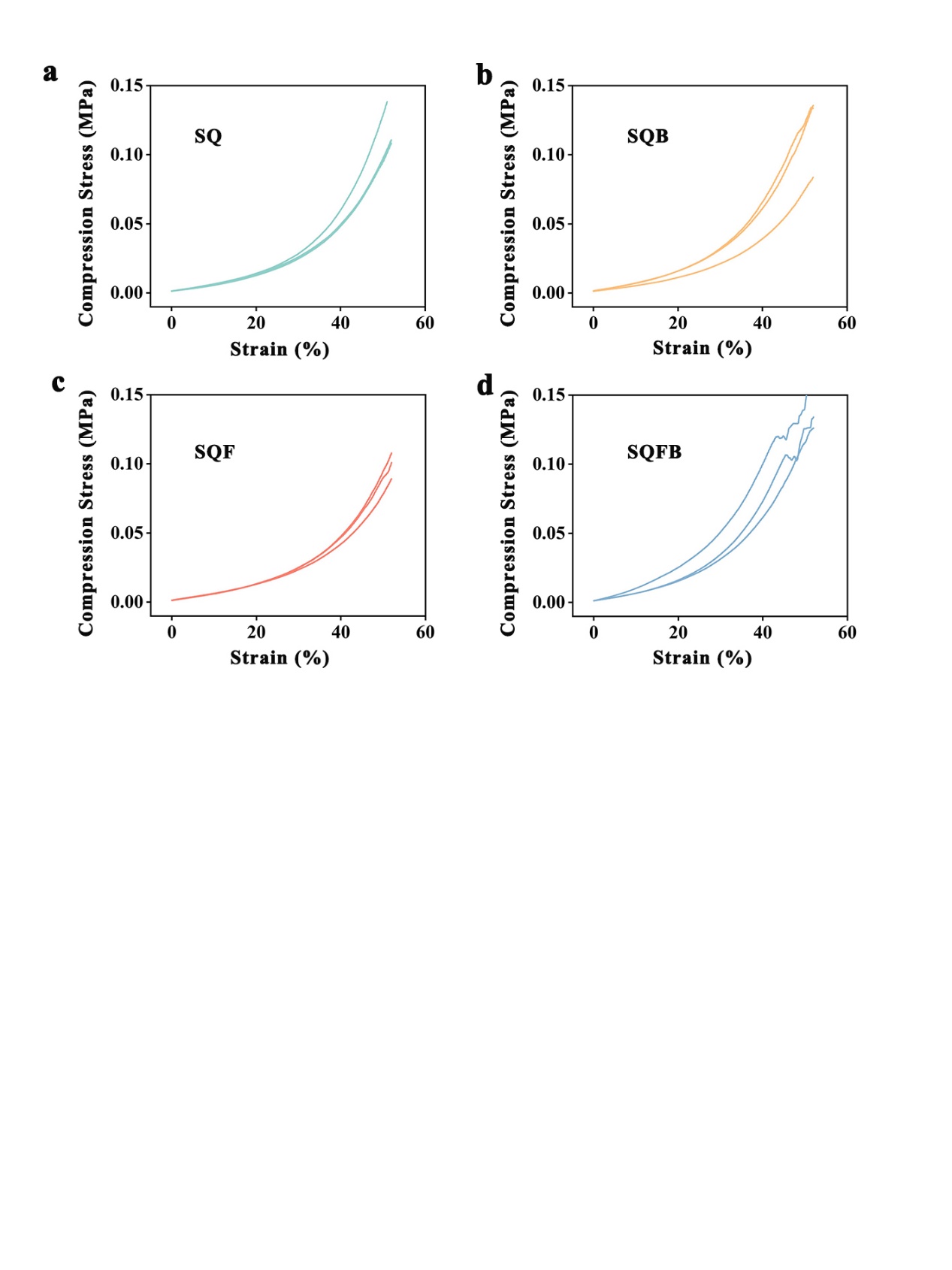


**Fig. S1** (a-d) Typical stress-strain curve of each group.


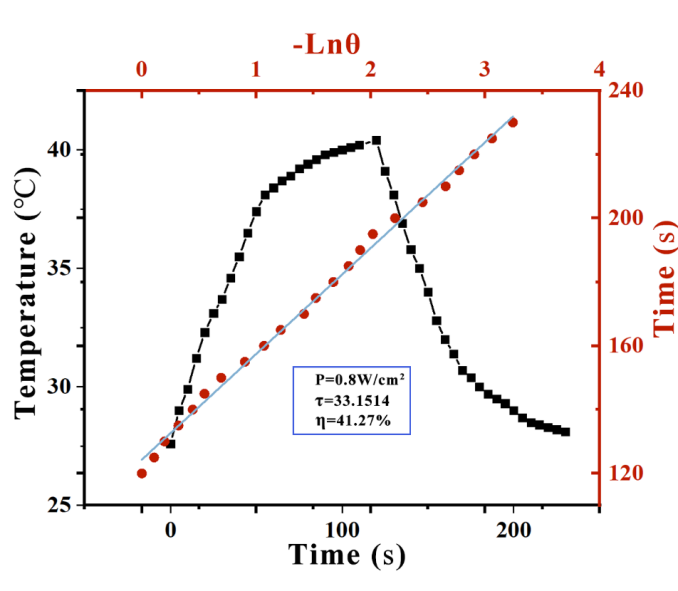


**Fig. S2** Photothermal conversion efficiency of SQFBs. Black line: photothermal effect of the SQFB under 808 nm NIR irradiation for 5 min; red line: time constant measured during cooling.


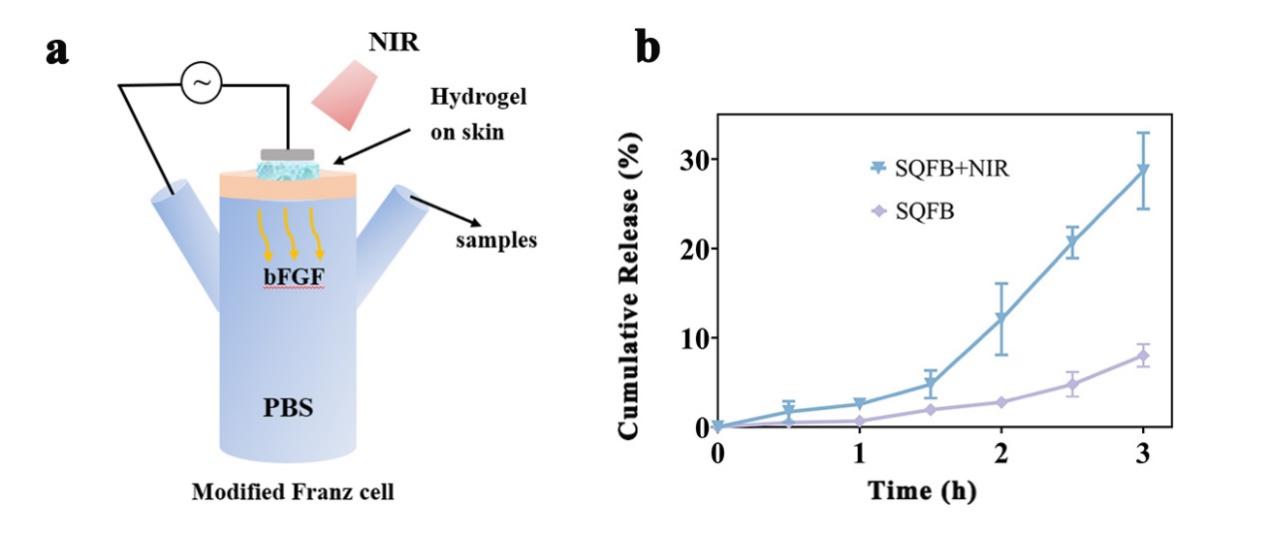


**Fig. S3** Characterization of SQFB for in vitro skin permeation drug release. (a) Modified Franz diffusion cell model. (b) Transdermal permeation curves of SQFB with and without NIR.


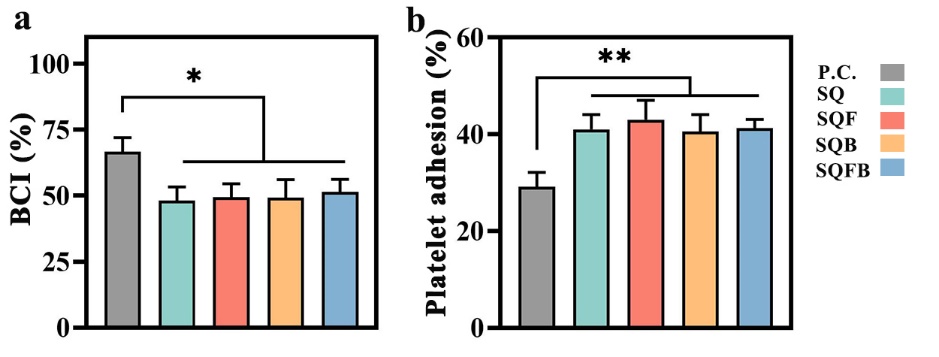


**Fig. S4** In vitro haemostatic performance of different hydrogels. (a) The BCI values of P.C., SQ, SQF, SQB and SQFB. (b) The platelet adhesion values of P.C., SQ, SQF, SQB and SQFB. Significant differences: *P < 0.05, **P < 0.01, and ***P < 0.001.


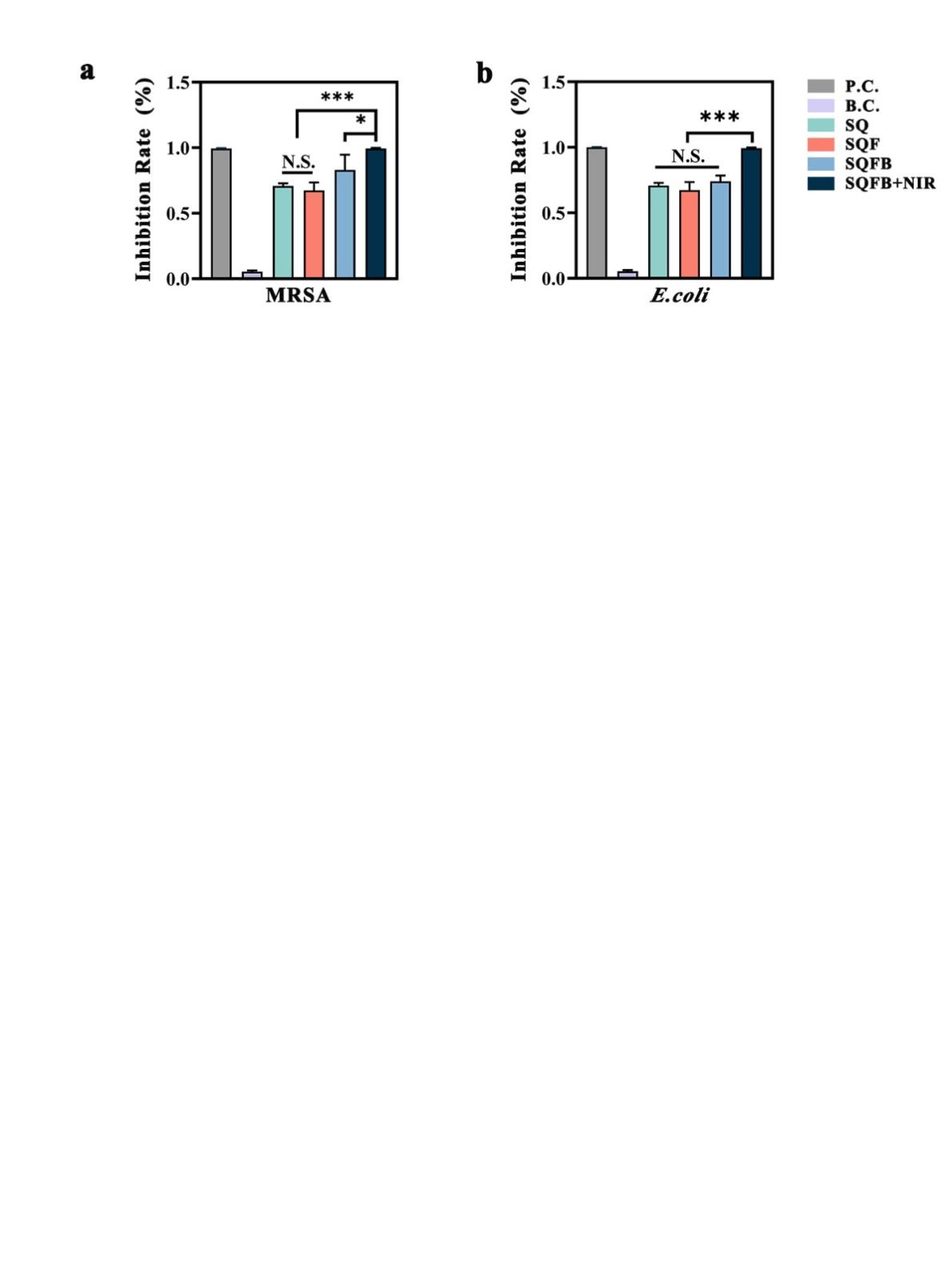


**Fig. S5** Quantitative analysis of inhibition rate by live/dead bacterial staining (a) for MRSA and (b) *E-coli*. Significant differences: *P < 0.05, **P < 0.01, and ***P < 0.001.


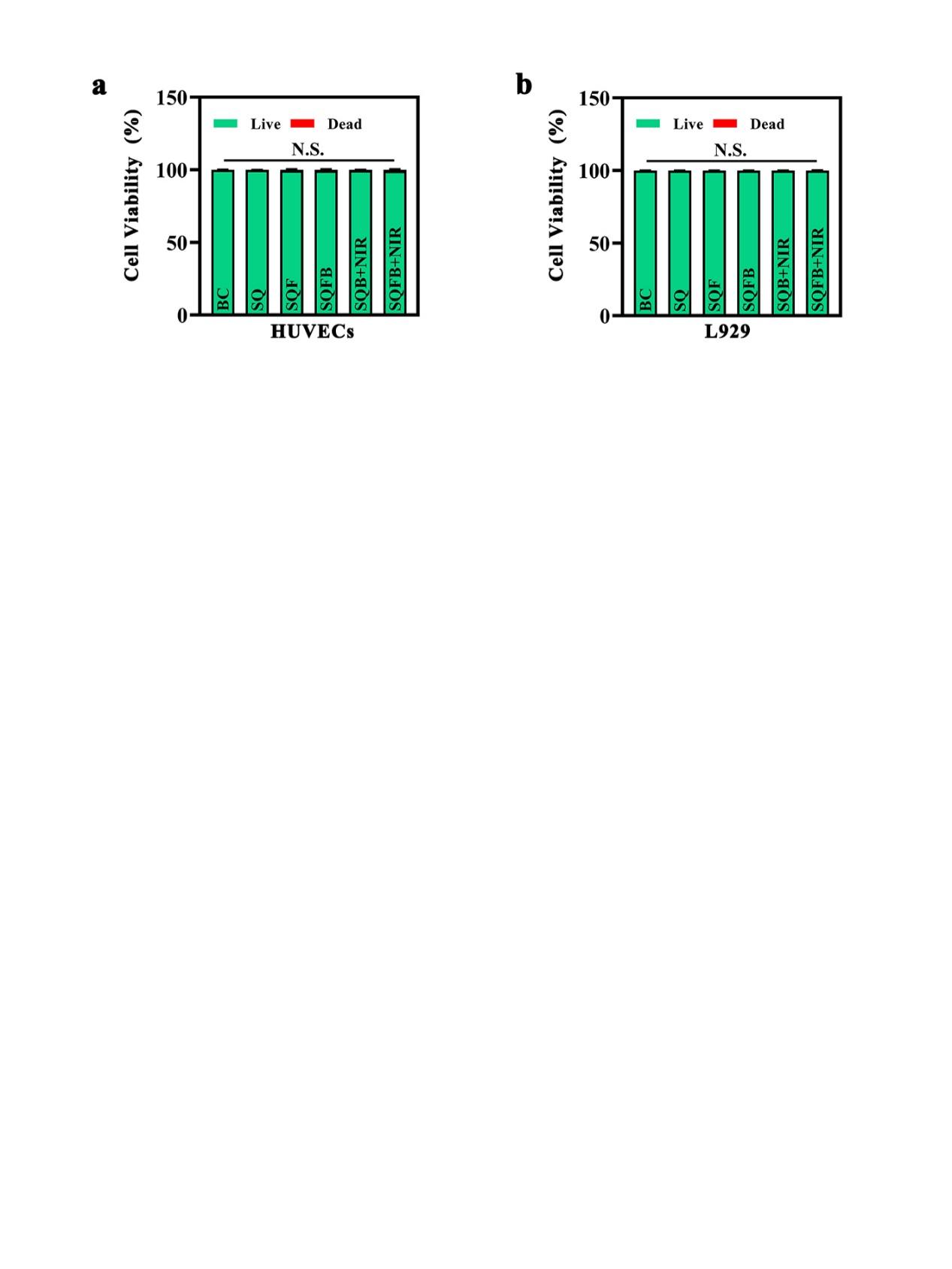


**Fig. S6** Quantitative analysis of live/dead cell staining (a) for HUVECs, (b) for L929.


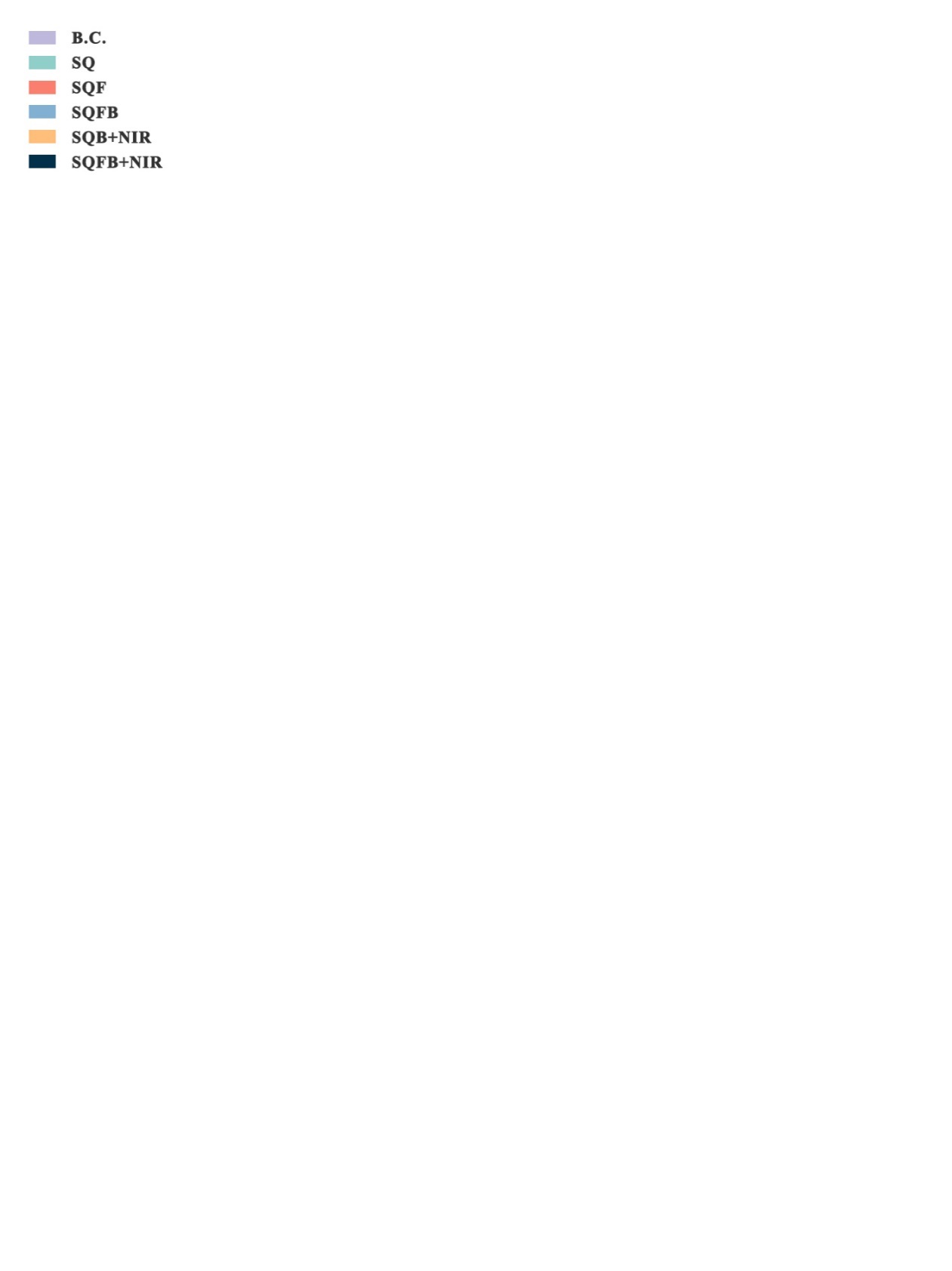

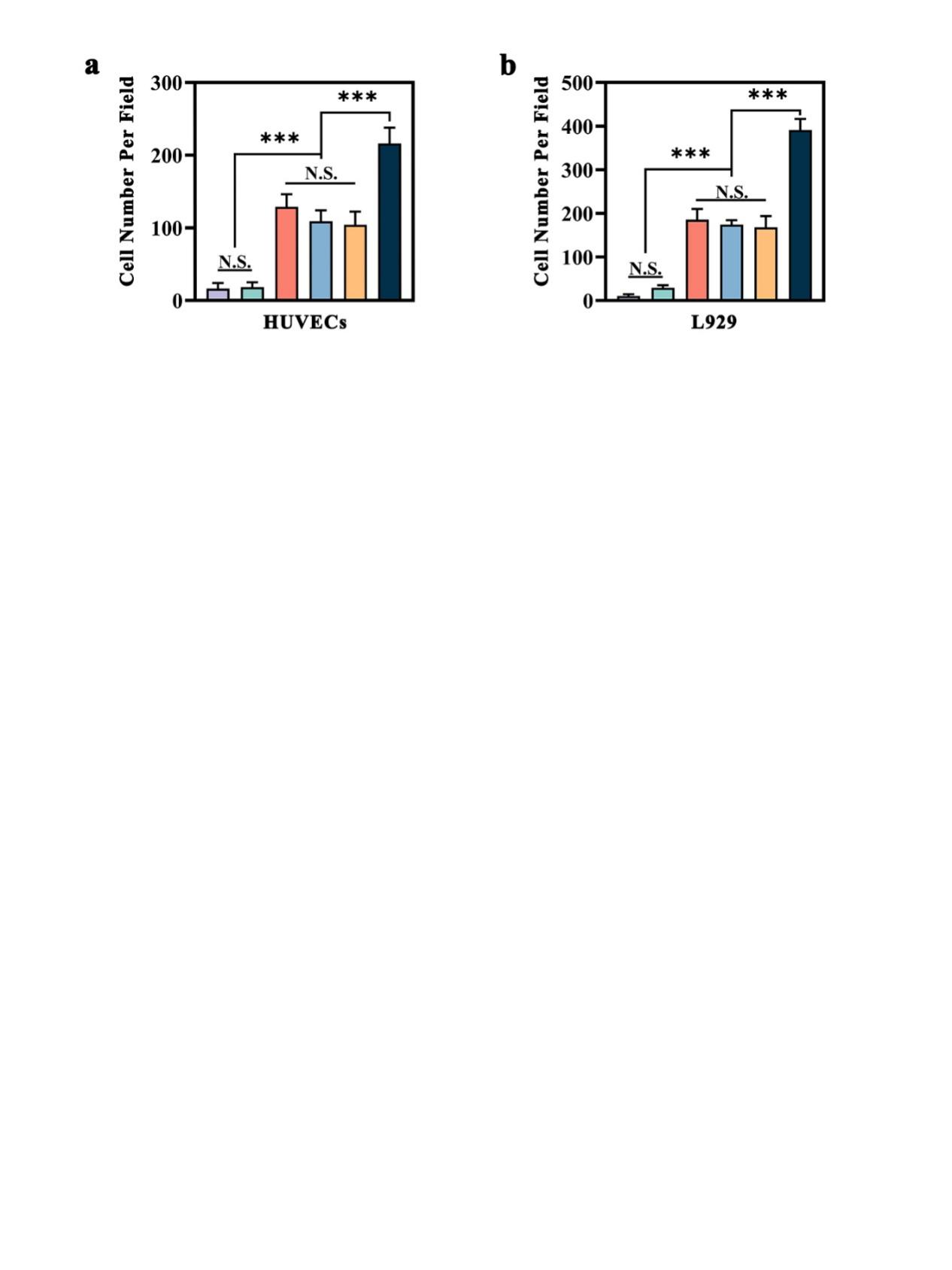


**Fig. S7** Quantitative analysis of cell number per field of the Transwell assay for (a) HUVECs, (b) L929, respectively. Significant differences: *P < 0.05, **P < 0.01, and ***P < 0.001.


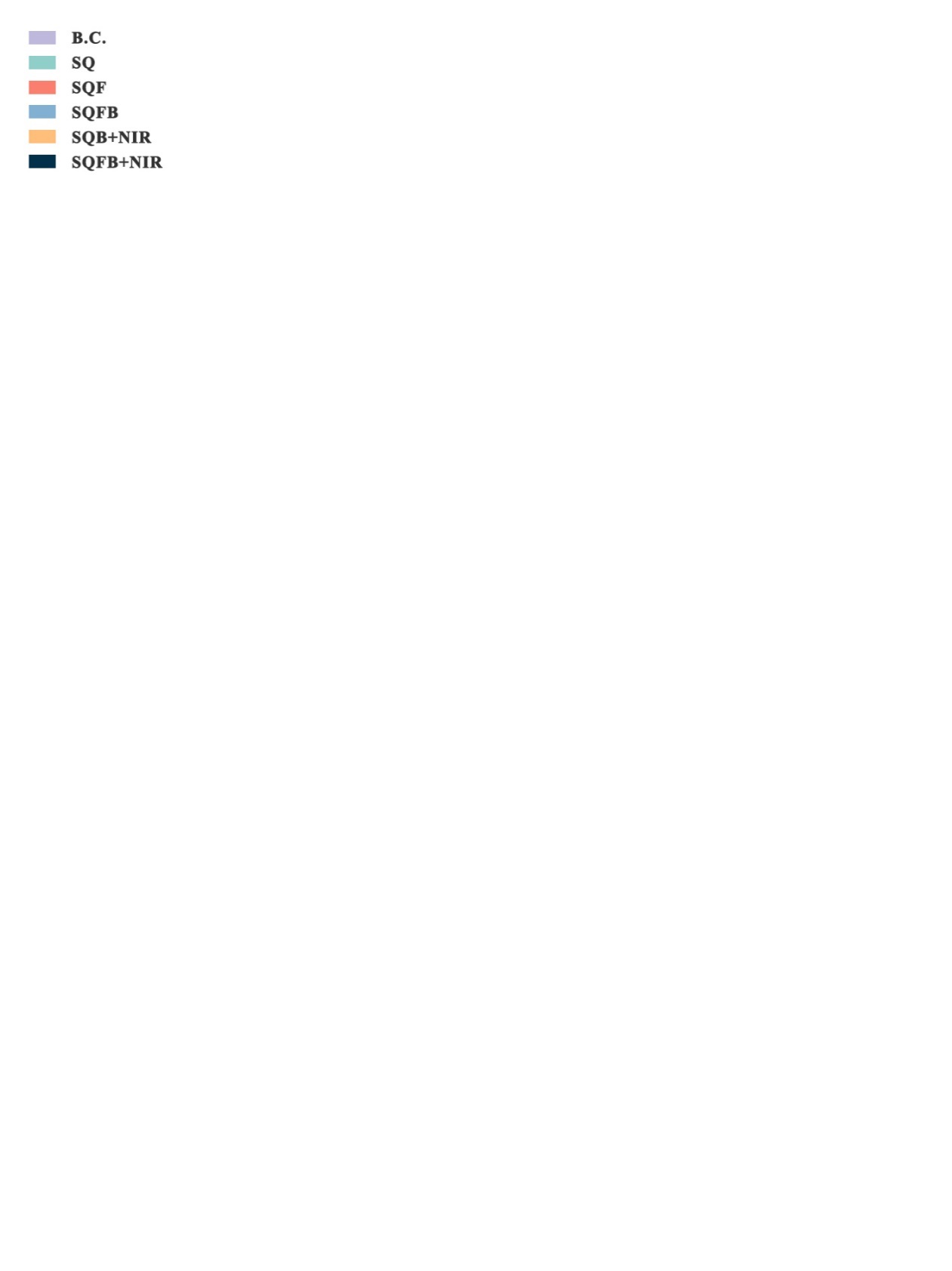

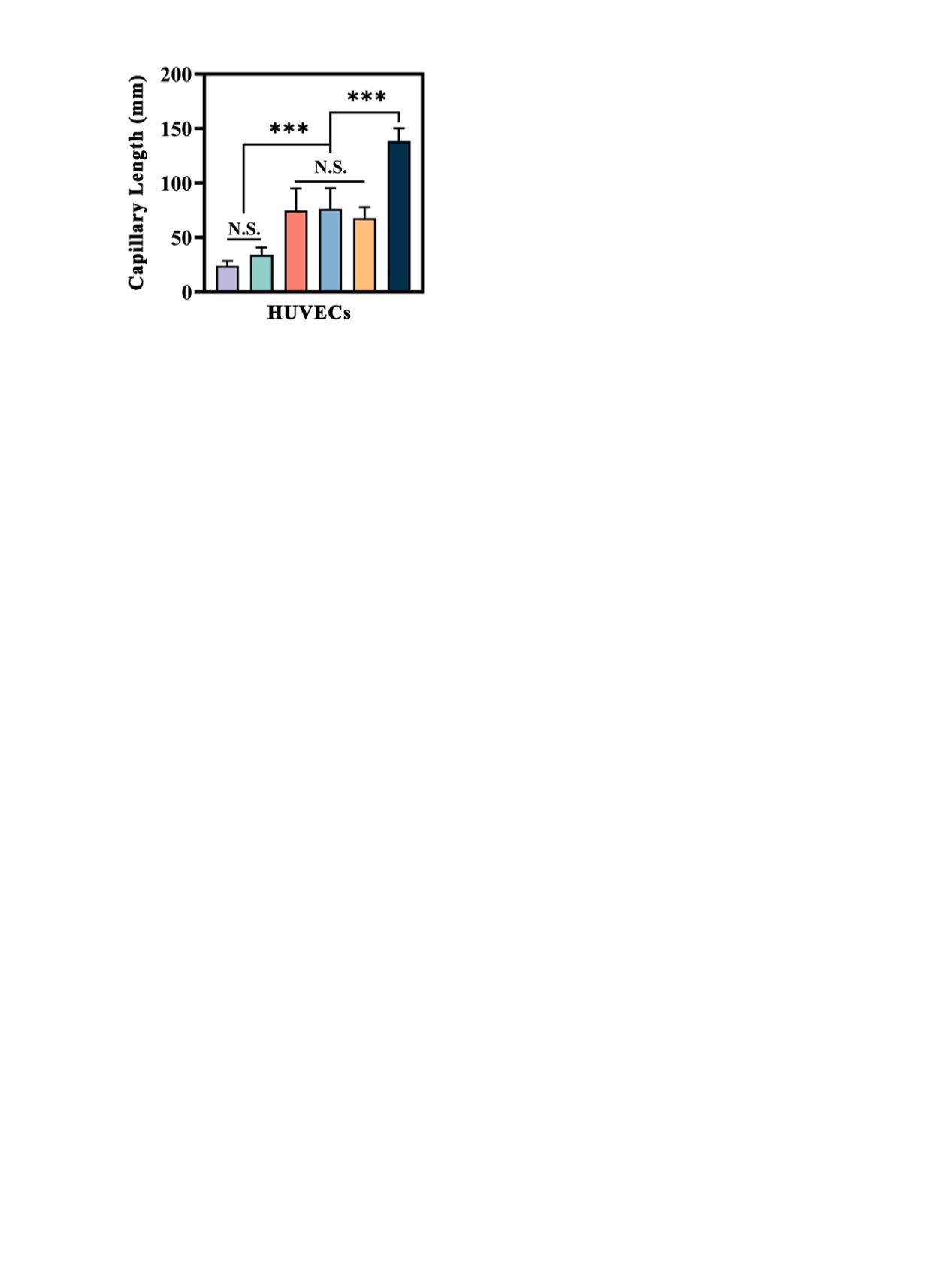


**Fig. S8** Quantification of cap length for tube formation assays. Significant differences: *P < 0.05, **P < 0.01, and ***P < 0.001.


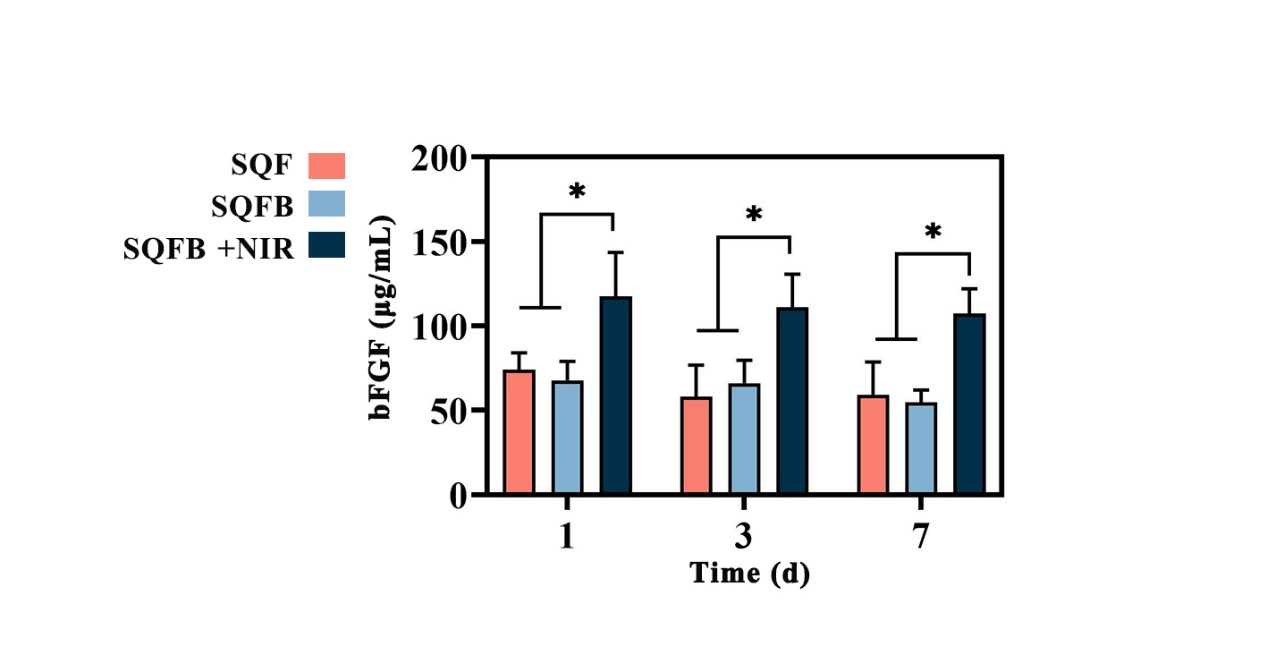


**Fig. S9** Concentrations of bFGF at wound sites in the skin at 1 d, 3 d and 7 d. Significant differences: *P < 0.05, **P < 0.01, and ***P < 0.001.


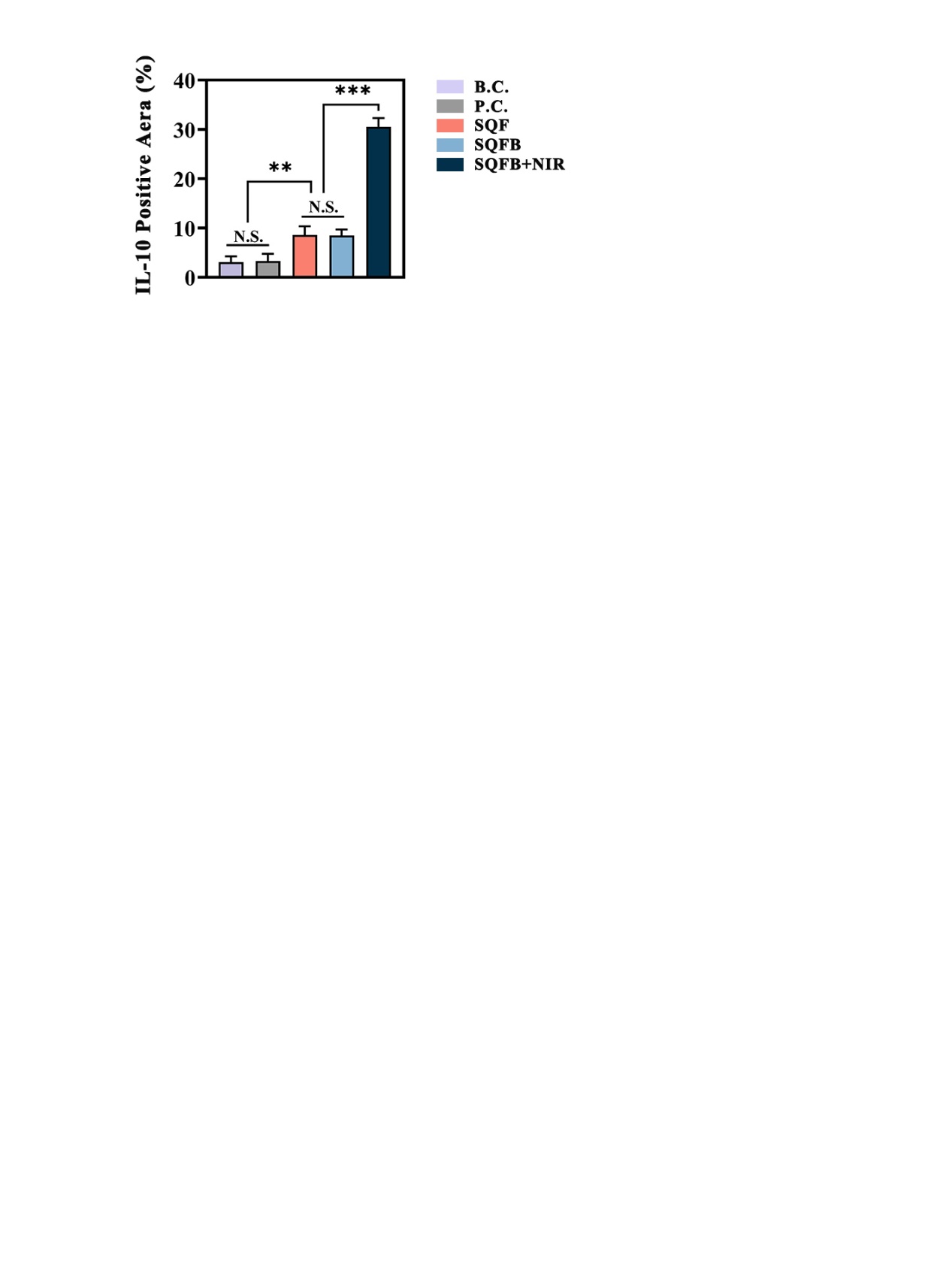


**Fig. S10** Quantitative statistics of IL-10 positive aera. Significant differences: *P < 0.05, **P < 0.01, and ***P < 0.001.


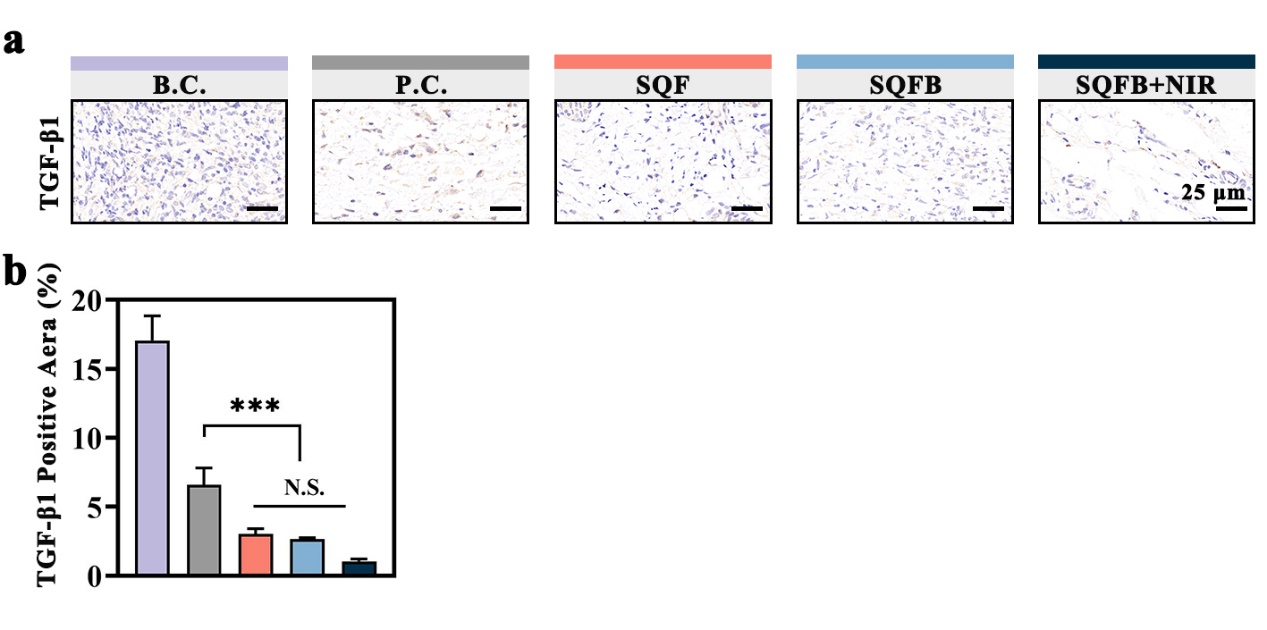


**Fig. S11** (a) Immunohistochemistry of TGF-β1 in different groups at day 12. (b) Quantitative statistics of TGF-β1 positive aera. Scale bar: 25 μm. Significant differences: *P < 0.05, **P < 0.01, and ***P < 0.001.


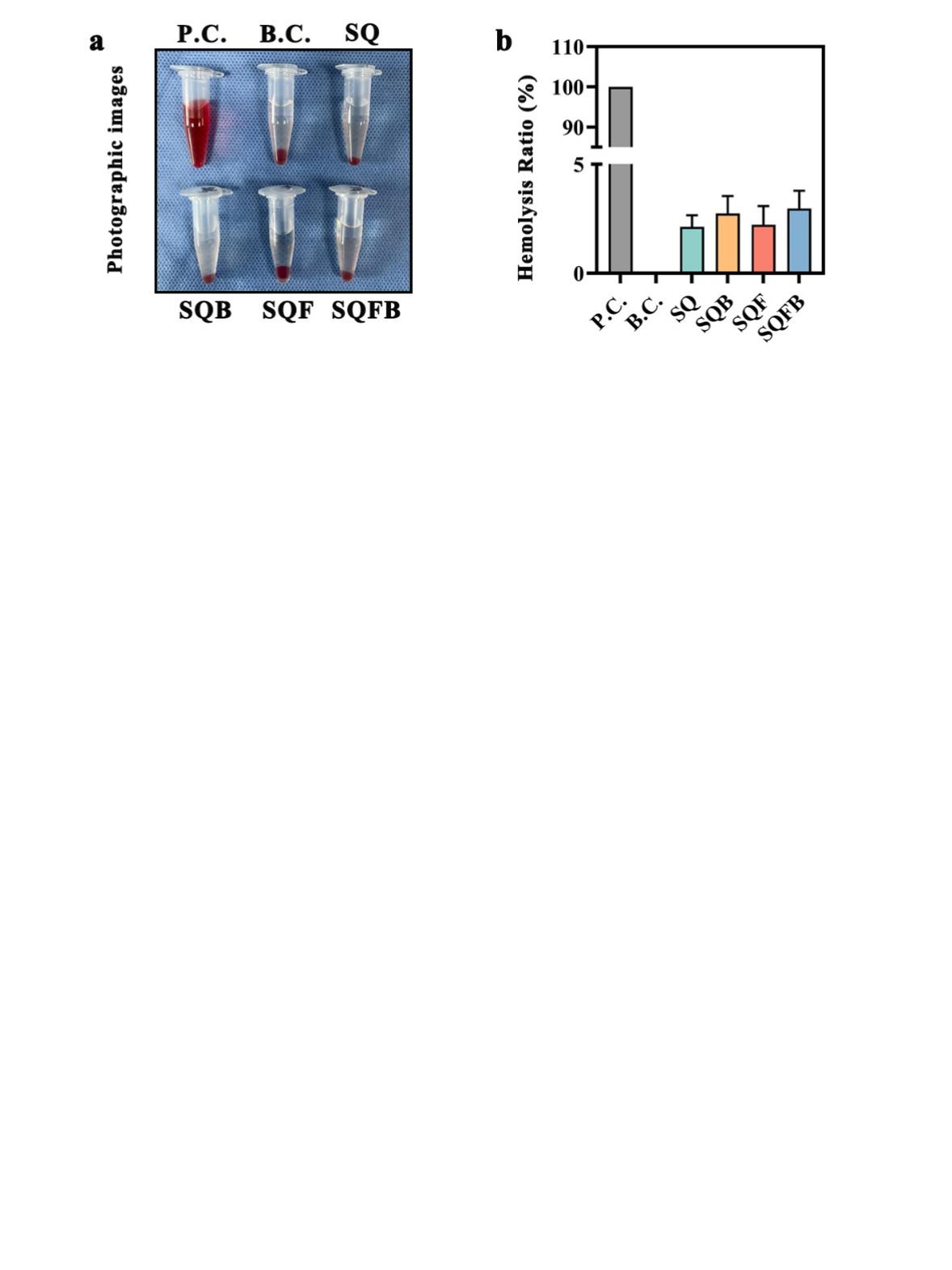


**Fig. S12** Blood compatibility testing of hydrogels. (a) Optical image of the hemolysis tests. (b) Hemolysis ratio of each group of hydrogels.


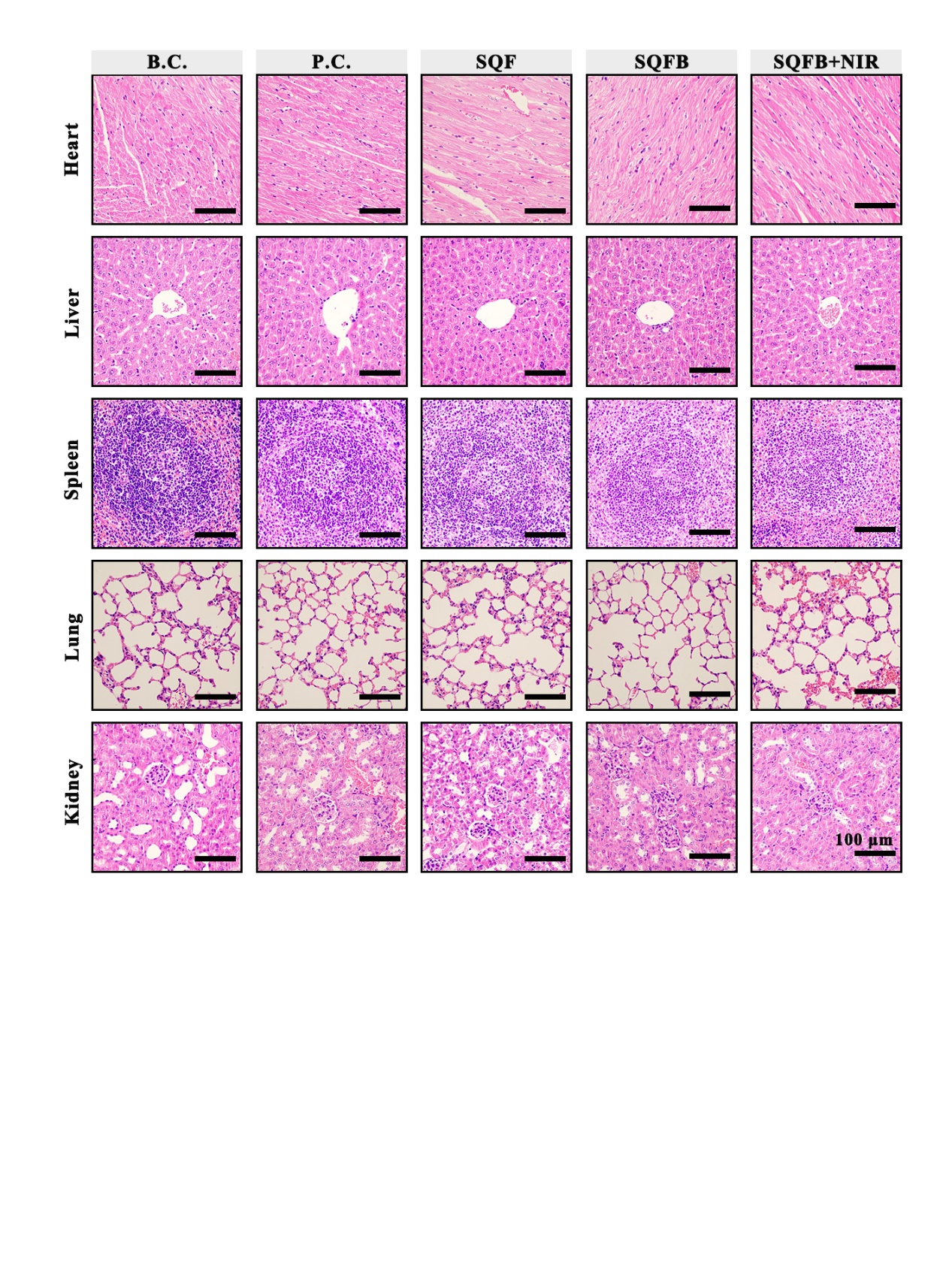


**Fig. S13** HE staining images of each group include heart, liver, spleen, lungs, and kidneys.


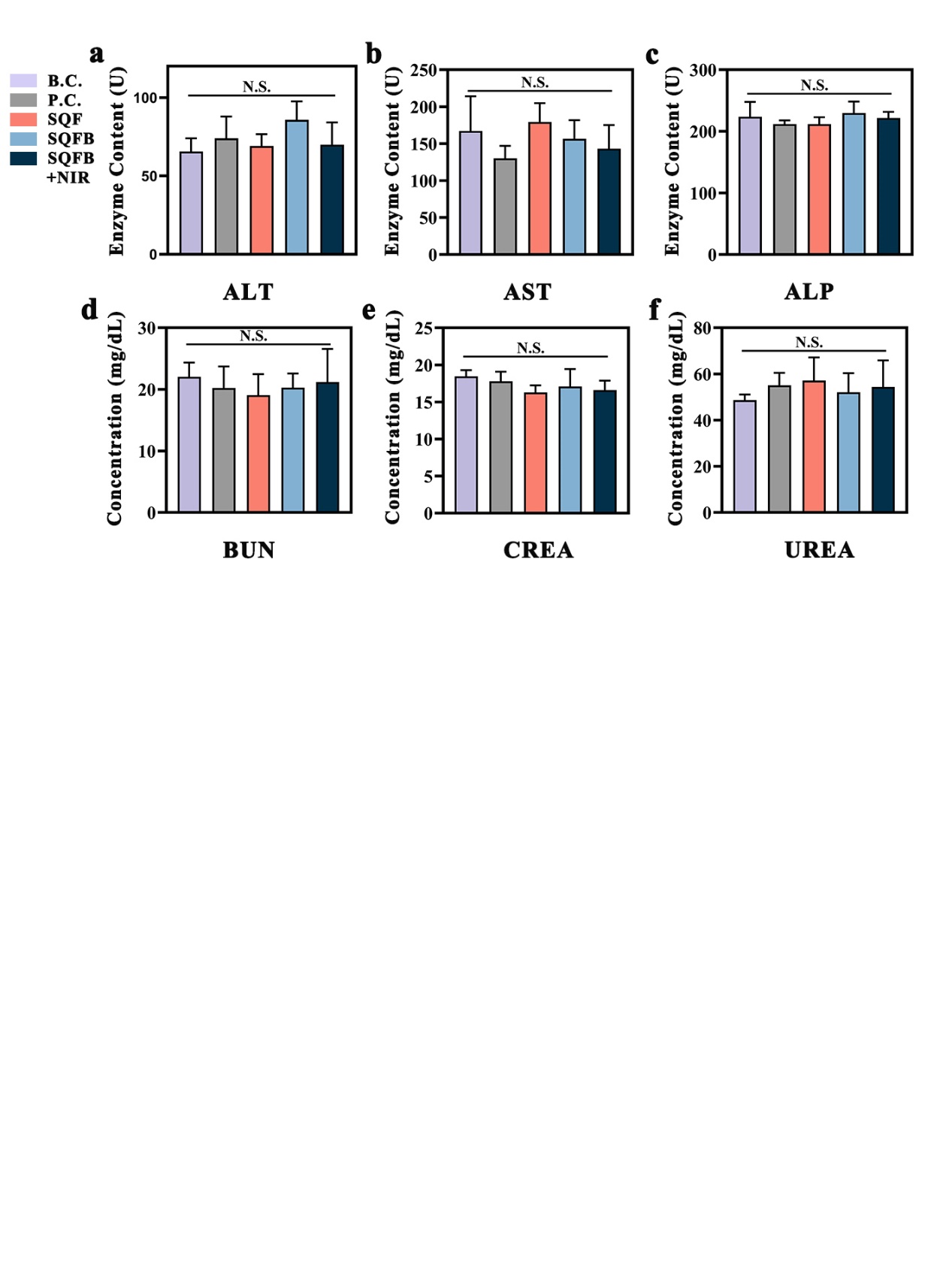


**Fig. S14** The results of biochemical tests in each group. (a-f) ALT, alanine aminotransferase; AST, aspartate aminotransferase; ALP, alkaline phosphatase; BUN, blood urea nitrogen; CREA, creatinine; UREA, urea.
